# Pivotal role of the muscle-contraction pathway in cryptorchidism and evidence for genomic connections with cardiomyopathy pathways in RASopathies

**DOI:** 10.1186/1755-8794-6-5

**Published:** 2013-02-14

**Authors:** Carlo V Cannistraci, Jernej Ogorevc, Minja Zorc, Timothy Ravasi, Peter Dovc, Tanja Kunej

**Affiliations:** 1 Integrative Systems Biology Laboratory, Biological and Environmental Sciences and Engineering Division, Computer, Electrical and Mathematical Sciences and Engineering Division, Computational Bioscience Research Center, King Abdullah University for Science and Technology (KAUST), Thuwal, Saudi Arabia; 2 Department of Mechanics, Politecnico di Torino, Turin, Italy; 3 Proteome Biochemistry Unit, San Raffaele Scientific Institute, Milan, Italy; 4 Department of Animal Science, Biotechnical Faculty, University of Ljubljana, Domzale, Slovenia

**Keywords:** Cryptorchidism, Muscle-contraction pathway, Cardiomyopathy, Comparative integratomics, Protein-protein interactions, Systems biology, Undescended testes, RASopathy

## Abstract

**Background:**

Cryptorchidism is the most frequent congenital disorder in male children; however the genetic causes of cryptorchidism remain poorly investigated. Comparative integratomics combined with systems biology approach was employed to elucidate genetic factors and molecular pathways underlying testis descent.

****Methods**:**

Literature mining was performed to collect genomic loci associated with cryptorchidism in seven mammalian species. Information regarding the collected candidate genes was stored in MySQL relational database. Genomic view of the loci was presented using Flash GViewer web tool (http://gmod.org/wiki/Flashgviewer/). DAVID Bioinformatics Resources 6.7 was used for pathway enrichment analysis. Cytoscape plug-in PiNGO 1.11 was employed for protein-network-based prediction of novel candidate genes. Relevant protein-protein interactions were confirmed and visualized using the STRING database (version 9.0).

****Results**:**

The developed cryptorchidism gene atlas includes 217 candidate loci (genes, regions involved in chromosomal mutations, and copy number variations) identified at the genomic, transcriptomic, and proteomic level. Human orthologs of the collected candidate loci were presented using a genomic map viewer. The cryptorchidism gene atlas is freely available online: http://www.integratomics-time.com/cryptorchidism/. Pathway analysis suggested the presence of twelve enriched pathways associated with the list of 179 literature-derived candidate genes. Additionally, a list of 43 network-predicted novel candidate genes was significantly associated with four enriched pathways. Joint pathway analysis of the collected and predicted candidate genes revealed the pivotal importance of the muscle-contraction pathway in cryptorchidism and evidence for genomic associations with cardiomyopathy pathways in RASopathies.

**Conclusions:**

The developed gene atlas represents an important resource for the scientific community researching genetics of cryptorchidism. The collected data will further facilitate development of novel genetic markers and could be of interest for functional studies in animals and human. The proposed network-based systems biology approach elucidates molecular mechanisms underlying co-presence of cryptorchidism and cardiomyopathy in RASopathies. Such approach could also aid in molecular explanation of co-presence of diverse and apparently unrelated clinical manifestations in other syndromes.

## Background

Cryptorchidism (CO) is the most frequent congenital disorder in male children (2-4% of full-term male births) and is defined as incomplete descent of one (unilateral) or both (bilateral) testes and associated structures. Cryptorchidism has a potential effect on health; defects in testes descent usually cause impaired spermatogenesis, resulting in reduced fertility and increased rates of testicular neoplasia, and testicular torsion (reviewed in [1]). Cryptorchidism is common in human, pigs, and companion animals (2–12%) but relatively rare in cattle, and sheep (≤ 1%) [2].

Testicular descent is a complex series of events which requires concerted action of hormones, constitutive mechanisms, and the nervous system. In most species, including human, the complete descent of testes usually occurs prenatally, while in some (*e.g.* dogs), postnatally. Beside environmental factors like endocrine disruptors, CO is at least in part determined by genetic causes (chromosome or gene mutations), and is often a common feature of different syndromes. For example, Klinefelter syndrome and mutations in *INSL3* gene have already been recognized as a cause of CO in some cases [3].

The comparative knowledge attained through study of animal models has been of great importance in understanding complex disease etiology, suggesting several candidate genes involved also in the pathogenesis of human diseases [4]. Therefore, the use of comparative genomics approach, integrating and cross-filtering the available knowledge from different species seems highly justified. Different animal models for CO exist; for example natural mutants or transgenic mice, rat, rabbit, dog, pig and rhesus monkeys are used to elucidate the role of different factors involved in CO [5]. Based on mouse knock-out models from Mouse Genome Informatics (MGI) database, several genes appear as possible candidates (*AR*, *HOX* genes, *INSL3*, *RXFP2*, and *WT1*). Additionally, the technological progress in the last years enabled the use of high-throughput omics-information, at coding (DNA), expression (RNA), and proteomic level. This technological revolution creates a vast amount of data, which increases the need for application of bioinformatics tools that are able to connect omics data with phenotype and enable search for overlapping pathogenetic mechanisms in different genetic diseases [6]. However, this existing technology hasn’t been significantly employed in human CO research on a genome and transcriptome-wide scale; to date only one genome-wide expression study has been performed in rat [7].

Integratomics represents a novel trend in the omics-research and is based on the integration of diverse omics-data (genomic, transcriptomic, proteomic, *etc.*), regardless of the study approach or species [8-10]. High genetic homology between mammals and the availability of well annotated genomes from different species allows the assembled data to be presented in a form of a comparative genomic view, displaying candidate genes as a single species orthologs.

Information extracted from diverse and methodologically focused studies are often fragmented and controversial. To overcome this problem we integrated the collected data, using a holistic (map-driven) approach, and developed freely available interactive genomic visualization tool. Such map-based approach allows identification and prioritization of candidate genes [11] based on a number of literature sources (references), genomic position, and pathway analyses, employing all currently available knowledge in different species. However, extrapolating the gained knowledge from one species to another is often difficult due to different anatomical and physiological characteristics, which should be considered when comparing pathology of the disease in different species.

To identify genetic factors potentially involved in CO pathogenesis in human we 1) applied comparative integratomics approach and assembled the database of all CO-associated genomic loci reported in the literature, regardless of the study approach and species, 2) presented the loci on a genomic map as human orthologs, and 3) prioritized the collected data using systems biology approach. The collected candidate genes were classified in corresponding biological pathways and the most significant CO-enriched pathways were proposed. Such classification of candidate genes allowed us to prioritize biological pathways (characterized by genes involved in the pathogenesis of CO), which revealed importance of several pathways (for example muscle contraction mechanisms) that may also play a role in the pathogenesis of other clinical features distinctive for different syndromes often concurrent with CO. In order to additionally illuminate the CO-associated pathways we performed a network-based protein-protein interaction analysis, which resulted in prediction of 43 additional CO candidate genes.

## Methods

In search for CO associated candidate loci seven different research approaches were considered: (i) chromosomal abnormalities associated with CO, (ii) copy number variations, (iii) clinical syndromes with known genetic mutations that feature CO, (iv) transgenes and knock-outs that result in CO associated phenotypes, (v) association studies/mutation screening that show association between sequence variation/mutation screening and CO, (vi) expression patterns associated with CO, and (vii) candidates associated with CO at proteomic level.

### Data mining

We reviewed the literature published up to 9/2012 searching for the relevant publications through PubMed (http://www.ncbi.nlm.nih.gov/pubmed/) and Web of Science (http://isiknowledge.com) using key phrases: genetics, gene candidates, cryptorchidism, testicular descent, undescended testes, male infertility, QTL, microarray, association, microRNA, non-coding RNA, epigenetic, reproduction, and assisted reproduction. CO-associated candidate genes from different sources and species were retrieved from the literature search. Human clinical syndromes that may cause or feature CO were retrieved from Online Mendelian Inheritance in Man (OMIM) database (http://www.ncbi.nlm.nih.gov/sites/entrez?db=omim) and Disease database (http://www.diseasesdatabase.com/). The data for CO-related experiments on mouse models were retrieved from the Mouse Genome Informatics (MGI) database (http://www.informatics.jax.org/). Human orthologs for the CO associated genes were extracted from the MGI database, which contains information about mammalian ortholog genes for different species. Overlap analysis of the CO candidate genes with genomic regions involved in chromosome mutations was performed using data retrieved from Ensembl via BioMart data mining tool.

### Database implementation

CO-associated candidate genes database is a web resource, which provides integrated and curated information on molecular components involved in the pathogenesis of CO. Information regarding collected CO-associated candidate genes has been stored in relational MySQL database, which is publicly available for search, data entry and update at http://www.integratomics-time.com/cryptorchidism/. Search interface enables users to find specific CO-associated candidate genes based on the number of criteria. Online data entry interface enables users to update or submit new CO-associated candidate genes.

### Genomic view of the CO associated loci

Overview of the chromosomal locations of CO associated loci is graphically represented in genomic view, as previously described [12]. It is possible to visualize the literature-collected and network-predicted CO genes on the same genomic view or separately. Genomic view is visible through the web-based interactive visualization tool Flash GViewer (http://gmod.org/wiki/Flashgviewer/), which was developed by the GMOD project.

### Pathway and network analysis

In the first pathway analysis we considered human orthologs of the literature-collected candidate genes (179 genes). DAVID Bioinformatics Resources 6.7 [13] was employed for the enrichment (overrepresentation) analysis. The background for the analysis was defined using the 179 candidate genes plus their first neighbours (5018 proteins) selected in the human protein-protein interaction network (PPIN). The result of the enrichment analysis was obtained using Bonferroni multiple test correction and a p-value significant threshold of 0.01. The human PPIN was obtained by fusion of the following human networks: IRefIndex [14], Chuang *et al.* article [15], Ravasi *et al.* article [16], Consensus-PathDB [17].

A new cohort of 43 candidate genes was predicted using PiNGO 1.11 [18]. PiNGO is a tool designed to find candidate genes in biological networks and it is freely provided as a plug-in for Cytoscape 2.8 [19], which is an open source software platform for visualizing and integrating molecular interaction networks. PiNGO predicts the categorization of a candidate gene based on the annotations of its neighbors, using enrichment statistics. In our analysis we quested which first-neighbour-genes significantly interact with the original cohort of 179 literature-collected genes in the human PPIN. We adopted: hypergeometric statistical test, Bonferroni multiple testing correction and p-value significant threshold of 0.01. The cohort of 43 network-predicted genes resulted strongly significant (Bonferroni p-value < 0.0095) for being new candidate genes.

In order to evaluate the importance of this new cohort of 43 candidate genes we performed the pathway analysis according to the procedure already described for the 179 literature-collected candidate genes.

Finally, in order to investigate the biological relations between the 179 literature-collected and 43 network-predicted genes, we repeated the pathway analysis in DAVID (using the same procedure previously described) considering the 222 (179 + 43) candidate genes. The background for the analysis was defined using the 222 candidate genes plus their first neighbours in the human PPIN. In addition, we visualized the protein-protein interactions occurring between the genes present in at least two pathways using the STRING database (version 9.0) [20] and selecting only interactions with high confidence score.

### Genetic variability of candidate genes

Genetic variability for the most promising CO candidate genes was extracted from the Ensembl database (http://www.ensembl.org/). Probably damaging genetic variations were predicted by PolyPhen-2, version 2.1.0, provided by Ensembl database. Putative polymorphic miRNA target sites in candidate genes were obtained from Patrocles database (http://www.patrocles.org/) [21].

## Results and discussion

**Figure 1:**
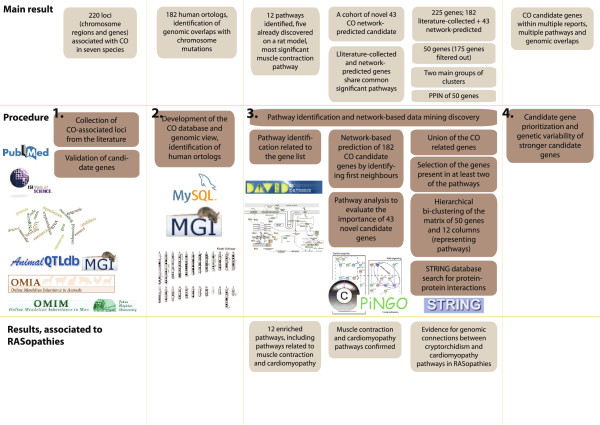
Workflow of the study.

Extensive literature mining was performed resulting in 217 collected candidate loci (chromosome regions and genes) reported to be involved in CO in human or/and animals. The generated database served as the foundation for the development of freely available interactive genomics viewer designed to integrate multi-species data from various research approaches. Enriched biological pathways and 43 additional CO candidate genes were suggested, based on protein-protein interaction network (PPIN) analysis. The workflow of the study is presented in the Figure 1.

### Collection of the cryptorchidism associated loci from the literature

The collected data incorporates genomic loci associated with cryptorchidism by seven different types of research approaches (chromosomal mutations, copy number variations, clinical syndromes, transgenes and knock-outs, association studies/mutation screening, transcriptomic/expression studies, and proteomic studies). The collected data originates from seven different species (human, cattle, horse, sheep, dog, rat, and mouse) (Table 1). The collected CO data is available in Additional file 1: Table S1, Additional file 2: Table S2, Additional file 3: Table S3, Additional file 4: Table S4 and Additional file 5: Table S5 and include physical locations of the candidate loci in human and species of origin.

**Table 1:** The summary of CO associated candidate loci

**Locus type / study approach**	**Number of loci**
*DNA level*	
Chromosomal aberrations	32
Copy number variants (CNVs)	2
Clinical syndromes	42
Knock-out and transgenic experiments	40
Association studies	12
*RNA level*	
Expression study	112
*Protein level*	
Injection of exogenous protein	1
**Total**	217*

* Unique loci (individual locus/gene reported by multiple studies was counted only once).

#### Chromosomal aberrations and copy number variations

We reviewed studies reporting 32 different chromosomal mutations including numerical and structural aberrations associated with cryptorchidism [22-33]. Additionally, two *de novo* copy number variations (CNVs) - microduplications were found to be associated with CO using array-based comparative genomic hybridization (aCGH) [34]. The collected data is available in Additional file 1: Table S1.

#### Clinical syndromes

Studies of complex disease traits can be facilitated by analysis of the molecular pathways represented by genes responsible for monogenic syndromes that also exhibit these traits [7,35]. There are over 200 different human syndromes with known molecular basis in OMIM database that feature “cryptorchidism” or “undescended testis” as a possible feature in their clinical synopsis. Since cryptorchidism phenotype prevalence is low in some syndromes, and could only occur coincidentally, it is difficult to justify association of syndrome causative genes with a particular phenotype.

To collect CO candidate genes (Additional file 2: Table S2) we obtained list of syndromes from the literature [4,36,37], OMIM and Diseases database (“may be caused or feature”) and then further examined phenotype-gene relationships and clinical features for each of the syndromes. Only syndromes where cryptorchidism is present as a regular feature, described in multiple clinical cases, and where gene(s) causing the syndrome is/are known were included.

#### Transgenes and knock-outs

From the Mouse Genome Informatics (MGI) database and the literature [38-42] we retrieved 39 mouse and one rat KO and transgenic experiments that result in phenotypes associated with CO (Additional file 3: Table S3).

#### Association studies/mutation screening

Nine genes (*AR*, *BMP7*, *ESR1*, *HOXA10*, *INSL3*, *KISS1R*, *NR5A1*, *RXFP2*, and *TGFBR3*) in human [43-59], *INSL3* in sheep [60] and dog [61], and *COL2A1* in dog [62] showed positive association between sequence polymorphisms/mutations and CO susceptibility (Additional file 4: Table S4). In the case of androgen receptor (*AR*) gene Ferlin *et al*. [45] found no difference between the numbers of CAG and GGC repeats, resulting in variable lengths of PolyGln/PolyGly in the *AR* gene and cryptorchidism; however, it has been proposed that a particular combination of the PolyGln/PolyGly polymorphisms may be linked to CO. In some cases opposing results have been found; for example, no association between the sequence polymorphisms and CO have been found for the genes *ESR1*[63-65], *INSL3*[66-68], *HOXA10*[69], and *RXFP2*[70]. The *LHCGR* has been excluded as a CO candidate gene in an association study in men [71], although KO of this gene in mice showed cryptorchid phenotype (MGI) and is causative gene of Leydig cell hypoplasia-a syndrome that features CO as one of the clinical signs (OMIM). In addition, Y chromosome microdeletions have been found to be present in patients with CO, but are not likely to be a common etiological cause of CO [72-74].

#### Expression patterns

There are several studies comparing expression profiles in testes between cryptorchid and normal males investigating the resulting effects of but not causes for development of CO (*e.g.*[75,76]). To our knowledge, there is only one microarray study that analyzed transcript profiles in gubernaculum during normal and abnormal testicular descent and reported 3589 differentially expressed genes between inherited cryptorchydism orl rats and a control group [7]. We included a subset of 112 promising candidate genes to our candidate gene list that were selected by the authors of the study based on expression levels, inclusion in specific pathways of interest and/or previous reports showing association with cryptorchidism (Additional file 5: Table S5).

#### Protein level

Hutson *et al.* (1998) [77] investigated the effect of exogenous calcitonin gene-related peptide (CGRP) in neonatal pigs. They found that exogenous CGRP, in pigs also known as calcitonin gene-related peptide B (CALCB), stimulated migration of inguinal testes that had been arrested in the line of descent, while ectopic testes did not respond. The results support the role for this protein in testicular descent. However, mutation screening performed by Zuccarello *et al*. (2004) [78] failed to confirm *CGRP* (in human also known as *CALCA*) pathway genes as a major players in human sporadic CO.

### Development of the CO database and genomic viewer

**Figure 2:**
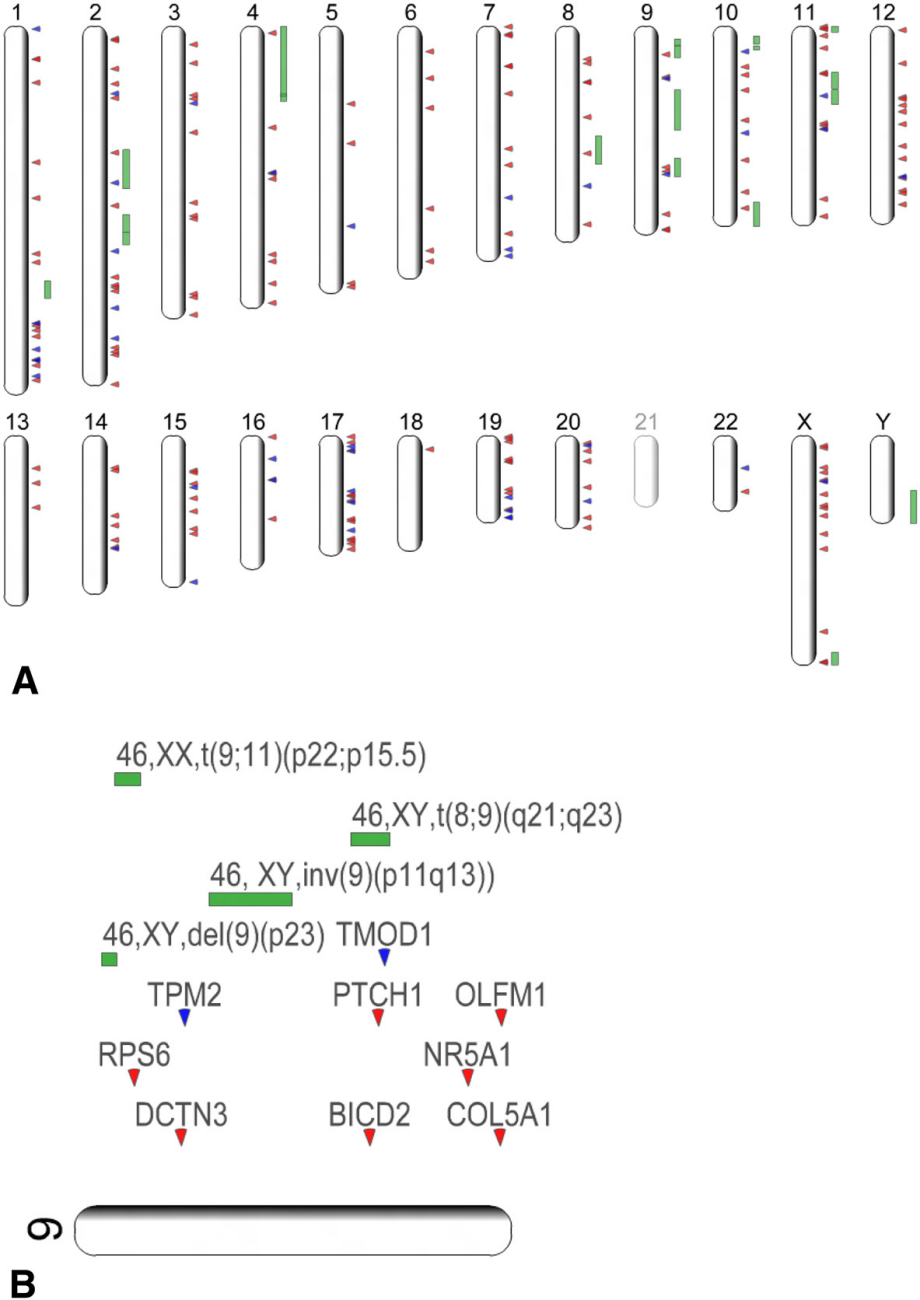
**Genomic view of the cryptorchidism candidate genes. A**. Genomic view of the literature-collected (red) and network-predicted (blue) CO associated candidate loci presented as human orthologs. The view includes syndromes with known genetic mutations that feature CO, mouse transgenic and knock-out experiments, chromosomal abnormalities, genes tested for association with CO, genes with expression patterns associated with CO, and genes associated with CO on proteomic level. Loci are placed at approximate positions on chromosome map. **B**. Enlargement of the chromosome 9.

The CO-associated loci, obtained by comparative integratomics approach, were assembled into a freely accessible database available at http://www.integratomics-time.com/cryptorchidism/. The curated database is open for public data entry. Researchers are invited to submit new cryptorchidism candidate genes from their research or other publications by filling data entry form on our web site. The collected loci from human and animal species were presented as genomic view for human orthologs (in a form of a human genomic view) (Figure 2).

Some candidate genes have been associated with CO by multiple independent literature reports in multiple species. For example, twenty genes (*AMH, AMHR2, AR, ARID5B, BMP7, EPHA4, ESR1, FGFR2, HOXA10, HRAS, INSL3, LHCGR, MAP2K1, MSX1*, *NR5A1, RXFP2, SOS1, TNNI2*, *TNNT3*, and *WT1*) have been associated with CO in at least two independent studies using different study approaches (Table 2). These genes are denoted in bold in the online database (http://www.integratomics-time.com/cryptorchidism/candidate_genes/).

**Table 2:** Literature-collected candidate genes associated with CO in at least two independent literature reports

**Gene**	**Clinical syndromes**	**KOs and transgenes**	**Overlapping chromosome mutations**	**Association studies (number of studies)**	**Expression experiments**
*AMH*	human	mouse			
*AMHR2*	human	mouse			
*AR*	human	mouse		human (2)	
*ARID5B*		mouse			rat
*BMP7*				human	rat
*EPHA4*		mouse			rat
*ESR1*		mouse		human (2)	
*FGFR2*	human		human		rat
*HOXA10*		mouse		human	rat
*HRAS*	human		human		rat
*INSL3*		mouse		human (5), sheep, dog	
*LHCGR*	human	mouse			
*MAP2K1*	human				rat
*MSX1*			human		rat
*NR5A1*		mouse		human	
*RXFP2*		mouse		human (4)	
*SOS1*	human				rat
*TNNI2*	human		human		
*TNNT3*	human		human		
*WT1*	human	mouse			

The CO associated loci mapped to all human chromosomes, except HSA21. Genomic distribution of the selected loci revealed several overlapping areas between the candidate loci. Overlaps between structural chromosomal mutations and candidate genes can be observed in Figure 2 or by using interactive genomic view available on the website (http://www.integratomics-time.com/cryptorchidism/genomic_view/). Genomic regions involved in chromosome mutations on chromosomes 2, 4, 8, 9, 11, and X [22-33] overlapped with 13 literature-collected candidate genes: *CAPG*, *MSX1*, *E2F5*, *PTCH1*, *BICD2*, *RPS6*, *FGFR2*, *HRAS*, *PAX6*, *WT1*, *TNNI2*, *TNNT3*, *FLNA*, and *MECP2*. For instance, a breakpoint on 11p15.5 overlapped with three CO candidate genes: *HRAS*, *TNNI2*, and *TNNT3.* Additionally, in some cases two regions involved in chromosome mutations overlapped; duplication on position 4p overlapped with *MSX1* gene and the region involved in chromosomal translocation on position 4p12. Interestingly, three network-predicted candidate genes, *FHL2, TMOD1* and *MYBPC3*, also overlapped with genomic regions involved in the chromosome mutations.

### Pathway identification and network-based data mining discovery

#### Pathway analysis of the cryptorchidism associated candidate genes

We performed pathway analysis of the 179 literature-collected CO-candidate genes (refer to Methods). This pathway enrichment analysis, conducted by applying very stringent criteria (Bonferroni multiple test correction and p-value significant threshold of 0.01), yielded the presence of twelve significant pathways associated with the list of our CO candidate genes in human (Table 3). The literature-collected candidate genes involved in multiple (at least four) pathways are presented in Additional file 6: Table S6 and marked with an asterisk in the online database.

**Table 3:** Pathway analysis of the literature-collected and network-predicted candidate genes, respectively

**Database**	**Pathway**	**Bonferonni p-value**	**Candidate genes involved in the pathway**
*Literature-collected candidate genes*			
KEGG	Regulation of actin cytoskeleton	4.70E-06	*ACTB, BRAF, CDC42, CFL1, CHRM3, EZR, FGD1, FGF9, FGFR1, FGFR2, HRAS, ITGB1, KRAS, MAP2K1, MAP2K2, MYL2, MYL9, PDGFA, PFN1, PPP1CA, PPP1CB, PXN, RAC1, RAF1, RHOA, RRAS, SOS1*
REACTOME	Muscle contraction	5.39E-06	*DES, MYH3, MYL2, MYL3, TNNI2, TNNT2, TNNT3, TPM1, TPM3, TPM4, TTN*
KEGG	Focal adhesion	1.05E-05	*ACTB, BRAF, CCND1, CDC42, COL1A2, COL2A1, COL5A1, FLNA, GRB2, GSK3B, HRAS, IGF1, ILK, ITGB1, MAP2K1, MYL2, MYL9, PDGFA, PPP1CA, PPP1CB, PXN, RAC1, RAF1, RHOA, SOS1, THBS4*
REACTOME	Signaling by PDGF	3.61E-5	*COL1A2, COL2A1, COL5A1, GRB2, HRAS, KRAS, MAP2K1, MAP2K2, PDGFA PTPN11, RAF1, SOS1, STAT3, THBS4*
REACTOME	Signaling by insulin receptor	7.02E-05	*EIF4E, EIF4EBP1, GRB2, HRAS, KRAS, MAP2K1, MAP2K2, RAF1, RPS6, RPS6KB1, SOS1*
REACTOME	Signaling by EGFR	0.0022	*CDC42, GRB2, HRAS, KRAS, MAP2K1, MAP2K2, PTPN11, PXN, RAF1, SOS1*
PANTHER	RAS pathway	0.0025	*BRAF, CDC42, GRB2, GSK3B, HRAS, KRAS, MAP2K1, MAP2K2, RAC1, RAF1, RHOA, RRAS, SOS1, STAT3*
KEGG	Hypertrophic cardiomyopathy (HCM)	0.0035	*ACTB, DES, IGF1, ITGB1, MYH7, MYL2, MYL3, TNNT2, TPM1, TPM3, TPM4, TTN*
BIOCARTA	Role of MAL in Rho-mediated activation of SRF	0.044	*ACTA1, CDC42, MAP2K1, MAP2K2, RAC1, RAF1, RHOA*
BIOCARTA	IGF-1 signaling	0.0067	*FOS, GRB2, HRAS, IGF1, MAP2K1, PTPN11, RAF1, SOS1*
PANTHER	Integrin signaling	0.0070	*BRAF, CDC42, COL1A2, COL2A1, COL5A1, FLNA, GRB2, HRAS, ILK, ITGB1, KRAS, MAP2K1, MAP2K2, PXN, RAC1, RAF1, RHOA, RND2, RRAS, SOS1*
KEGG	Dilated cardiomyopathy	0.0099	*ACTB, DES, IGF1, ITGB1, MYH7, MYL2, MYL3, TNNT2, TPM1, TPM3, TPM4, TTN*
*Network-predicted candidate genes*			
REACTOME	Muscle contraction	2.71E-24	*ACTN2, DMD, MYBPC1, MYBPC2, MYBPC3, MYH8, MYL1, MYL4, NEB, TCAP, TMOD1, TNNC1, TNNC2, TNNI1, TNNI3, TNNT1, TPM2, VIM*
KEGG	Hypertrophic cardiomyopathy (HCM)	9.68E-6	*ACTC1, DMD, MYBPC3, TGFB1, TGFB2, TGFB3, TNNC1, TNNI3, TPM2,*
PANTHER	TGF-beta signaling pathway	1.07E-5	*BMP2, LEFTY1, LEFTY2, LOC100271831, MAPK1, MAPK3, MSTN, NODAL, TGFB1, TGFB2, TGFB3*
KEGG	Dilated cardiomyopathy	2.35E-5	*ACTC1, DMD, MYBPC3, TGFB1, TGFB2, TGFB3, TNNC1, TNNI3, TPM2*

The presence of pathways related to “cytoskeleton”, “muscle development”, “muscle contraction”, “focal adhesion”, and “insulin signaling” was previously reported in rat [7]. In addition to these pathways, our analysis showed new pathways: “cardiomyopathy” (hypertrophic and dilated),“RAS signaling”, “signaling by PDGF”, “signaling by EGFR”, “role of MAL in Rho-mediated activation of SRF”, “IGF-1 signaling pathway”, and “integrin signaling”. The results represent a valid example of pathway-based data mining discovery.

As an additional validation analysis, we excluded the 112 candidate genes proposed by Barthold *et al*. (2008) [7] from the overall candidate genes list (consisting of 179 unique human genes) and repeated the pathway analysis. Nine genes from Barthold *et al*. (2008) [7] were reported as CO candidate genes also in other studies, therefore we retained them in the analysis, so that the new list of candidate genes consisted of 79 genes. The pathway analysis of these remaining 79 candidate genes returned similar results as were obtained when using the overall 179 candidate gene list. In fact, 10 of the 12 enriched pathways were the same after excluding the discussed data from the candidate gene list. In particular, the five pathways reported by Barthold *et al*. (2008) [7] in rat (“cytoskeleton”, “muscle development”, “muscle contraction”, “focal adhesion”, and “insulin signaling”) were all confirmed in this independent validation analysis. The main effect of the gene removal were higher, but still significant, p-values in the pathway analysis. According to these results we can infer that inclusion of the candidate genes from Barthold *et al*. (2008) [7] is not the reason for the substantial overlap of the five pathways identified in both studies. On the contrary, the findings proposed here are a further confirmation of the validity of the conclusions made by Barthold *et al*. (2008) [7].

Surprisingly, when we searched the medical literature for articles that describe pathologies where CO, cardiomyopathy, and RAS signaling are common features, we found a perfect matching with Noonan, Cardiofaciocutaneous, LEOPARD, and Costello syndrome that all belong to the class of RASopathies [79,80]. Features of all four syndromes are different physical anomalies including concomitant presence of cardiomyopathy due to heart defects and, in males, cryptorchidism [79]. Noonan syndrome (NS) is the most common single gene cause of congenital heart disease, and NS subjects also present other features as leukemia predisposition [81]. In particular, five different mutations in *RAF1* were identified in individuals with NS; four mutations causing changes in the CR2 domain of RAF1 were associated with hypertrophic cardiomyopathy (HCM), whereas mutations in the CR3 domain were not [82]. Additionally, *PTPN11*, *RAF1*, and *SOS1* mutants were identified as a major cause of Noonan syndrome, *BRAF* of Cardiofaciocutaneous, *PTPN11* of LEOPARD, and *HRAS* of Costello syndrome, providing new insights into RAS regulation [80,81]. These genes have also been found to be mutated in patients with RASopathies having cryptorchidism in a clinical picture. In NS patients having CO in their clinical picture 11/14 had mutated *PTPN11*, 4/5 had mutated *SOS1*, and 1/2 had mutated *RAF1*. *BRAF* has been found to be mutated in 2/3 patients with Cardiofaciocutaneous syndrome having CO, *PTPN11* in 1/4 patients with LEOPARD having CO, and *HRAS* in 2/4 patients with Costello syndrome and CO [80,81]. However, the genes responsible for the remainder are unknown, and the gene pathway relations responsible for potential connections between unrelated features such as cryptorchidism and HCM in RASopathies are not clear. Therefore, we performed a network-based prediction (see next paragraph) of CO candidate genes by identifying the most significant first neighbors (in the human protein-protein interaction network; PPIN) of the 179 literature-collected candidates.

#### Pathway analysis of the network-predicted candidate genes

A new cohort of 43 candidate genes (Additional file 7: Table S7) was predicted by PiNGO 1.11 [18], which is a Cytoscape plug-in (see Methods) [19]. The question we tried to address was which first-neighbor genes significantly interact with the original cohort of 179 literature-collected genes in the human PPIN. We adopted hypergeometric statistical test and Bonferroni multiple testing correction. The cohort of 43 network-predicted genes was strongly significant (Bonferroni p-value < 0.0095); therefore, we consider them as additional CO candidate genes.

In order to evaluate the importance of these new candidate genes we performed the pathway analysis (Table 3), according to the same procedure already used in the previous paragraph (and described in the methods). The most intriguing evidence is the presence of significant pathways related to cardiomyopathy and muscle contraction in both sets of candidate genes (*i.e.* literature-collected and network-predicted). Pathways common to both sets of candidate genes represent a confirmation of the validity and robustness of the results obtained in the first pathway analysis and regarding the hypothesis of connection between CO and cardiomyopathy, in NS. Yet, it is also a quality proof of the procedure adopted for network prediction of new candidate genes.

#### Pathway analysis of the overall CO candidate gene list (179 literature-collected and 43 network-predicted genes)

The first cohort of 179 literature-collected genes and the second one containing 43 network-predicted genes were condensed in a list of 222 unique genes - the overall candidate gene list. We repeated the pathway analysis on this list applying the same very stringent criteria used above (Bonferroni multiple test correction and p-value significant threshold of 0.01). The analysis suggested the presence of 12 significant pathways associated with the overall list of candidate genes in human (Table 4).

**Table 4:** Pathway analysis of the overall candidate gene list (literature-collected and network-predicted)

**Database**	**Pathway**	**Bonnferoni p-value**	**Candidate genes involved in the pathway**
REACTOME	Muscle contraction	4.55E-33	*ACTN2, DES, DMD, MYBPC1, MYBPC2, MYBPC3, MYH3, MYH8, MYL1, MYL2, MYL3, MYL4, NEB, TCAP, TMOD1, TNNC1, TNNC2, TNNI1, TNNI2, TNNI3, TNNT1, TNNT2, TNNT3, TPM1, TPM2, TPM3, TPM4, TTN, VIM*
KEGG	Hypertrophic cardiomyopathy (HCM)	1.21E-9	*ACTB, ACTC1, DES, DMD,IGF1, ITGB1, MYBPC3, MYH7, MYL2, MYL3, TGFB1, TGFB2, TGFB3, TNNC1, TNNI3, TNNT2, TPM1, TPM2, TPM3, TPM4, TTN*
KEGG	Dilated cardiomyopathy	1.21E-8	*ACTB, ACTC1, DES, DMD, IGF1, ITGB1, MYBPC3, MYH7, MYL2, MYL3, TGFB1, TGFB2, TGFB3, TNNC1, TNNI3, TNNT2, TPM1, TPM2, TPM3, TPM4, TTN*
KEGG	Focal adhesion	4.68E-7	*ACTB, ACTN2, BRAF, CAV1, CCND1, CDC42, COL1A2, COL2A1, COL5A1, FLNA, GRB2, GSK3B, HRAS, IGF1, IGF1R, ILK, ITGB1, MAP2K1, MAPK1, MAPK3, MYL2, MYL9, PDGFA, PPP1CA, PPP1CB, PRKCA, PXN, RAC1, RAF1, RHOA, SOS1, THBS4*
REACTOME	Signaling by insulin receptor	3.83E-6	*EIF4E, EIF4EBP1, GRB2, HRAS, KRAS, MAP2K1, MAP2K2, MAPK1, MAPK3, RAF1, RHEB, RPS6, RPS6KB1, SOS1*
KEGG	Regulation of actin cytoskeleton	4.29E-6	*ACTB, ACTN2, BRAF, CDC42, CFL1, CHRM3, EZR, FGD1, FGF3, FGF9, FGFR1, FGFR2, HRAS, ITGB1, KRAS, MAP2K1, MAP2K2, MAPK1, MAPK3, MYL2, MYL9, PDGFA, PFN1, PPP1CA, PPP1CB, PXN, RAC1, RAF1, RHOA, RRAS, SOS1*
PANTHER	TGF-beta signaling pathway	7.01E-6	*AMH, AMHR2, BMP2, BMP4, BMP5, BMP7, CDC42, FOS, FOXO1, FOXP3, HRAS, KRAS, LEFTY1, LEFTY2, MAPK1, MAPK3, MSTN, NODAL, RHEB, RRAS, TGFB1, TGFB2, TGFB3*
BIOCARTA	Integrin signaling pathway	1.35E-5	*ACTA1, ACTN2, CAV1, GRB2, HRAS, ITGB1, MAP2K1, MAP2K2, MAPK1, MAPK3, PXN, RAF1, RHOA, SOS1*
REACTOME	Signaling by PDGF	7.27E-5	*COL2A1, COL1A2, COL5A1, GRB2, HRAS, KRAS, MAP2K1, MAP2K2, MAPK1, MAPK3, PDGFA, PTPN11, RAF1, SOS1, STAT3, THBS4*
KEGG	Cardiac muscle contraction	0.0024	*ACTC1, MYH7, MYL2, MYL3, TNNC1, TNNI3, TNNT2, TPM1, TPM2, TPM3, TPM4*
PANTHER	RAS pathway	0.0030	*BRAF, CDC42, GRB2, GSK3B, HRAS, KRAS, MAP2K1, MAP2K2, MAPK1, MAPK3, RAC1, RAF1, RHOA, RRAS, SOS1, STAT3*
KEGG	Vascular smooth muscle contraction	0.0058	*ACTA2, BRAF, MAP2K1, MAP2K2, MAPK1, MAPK3, MYH11, MYL9, PPP1CA, PPP1CB, PRKCA, PRKCE, RAF1, RHOA*

**Figure 3:**
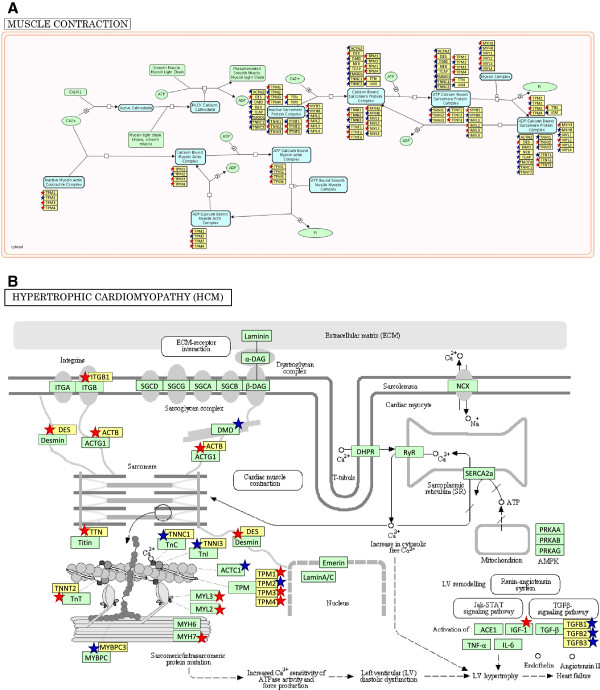
**Pathways, containing literature-collected and network-predicted CO candidate genes.** CO genes are marked with stars: red stars stand for literature-collected genes, blue stars stand for network-predicted genes. Gene names are added in yellow boxes to the original pathway images. **A**. Reactome pathway: Muscle contraction – human. **B**. KEGG pathway: Hypertrophic cardiomyopathy (HCM) – human.

The “muscle contraction” pathway was the most significant (in absolute) with Bonferroni corrected p-value of 4.55E-33 (Figure 3A); while the “hypertrophic cardiomyopathy” was the second most significant pathway with Bonferroni corrected p-value of 1.21E-09 (Figure 3B). These results are crucial for our study because they suggest the presence of a strong genomic connection among diverse pathways associated with clinical features that seemed unrelated. To address relationship among these mechanisms we created a matrix merging the information related to the gene participation in several identified pathways. Of the 222 (179 + 43) candidate genes, 172 were filtered out because they were not present in at least two of the 12 significant pathways. The resulting matrix consists of 50 candidate genes in the rows and 12 enriched pathways in the columns (Additional file 8: Table S8). The matrix values are binary: 0 indicates that the gene is not present in a pathway, whereas 1 indicates that the gene is present.

**Figure 4:**
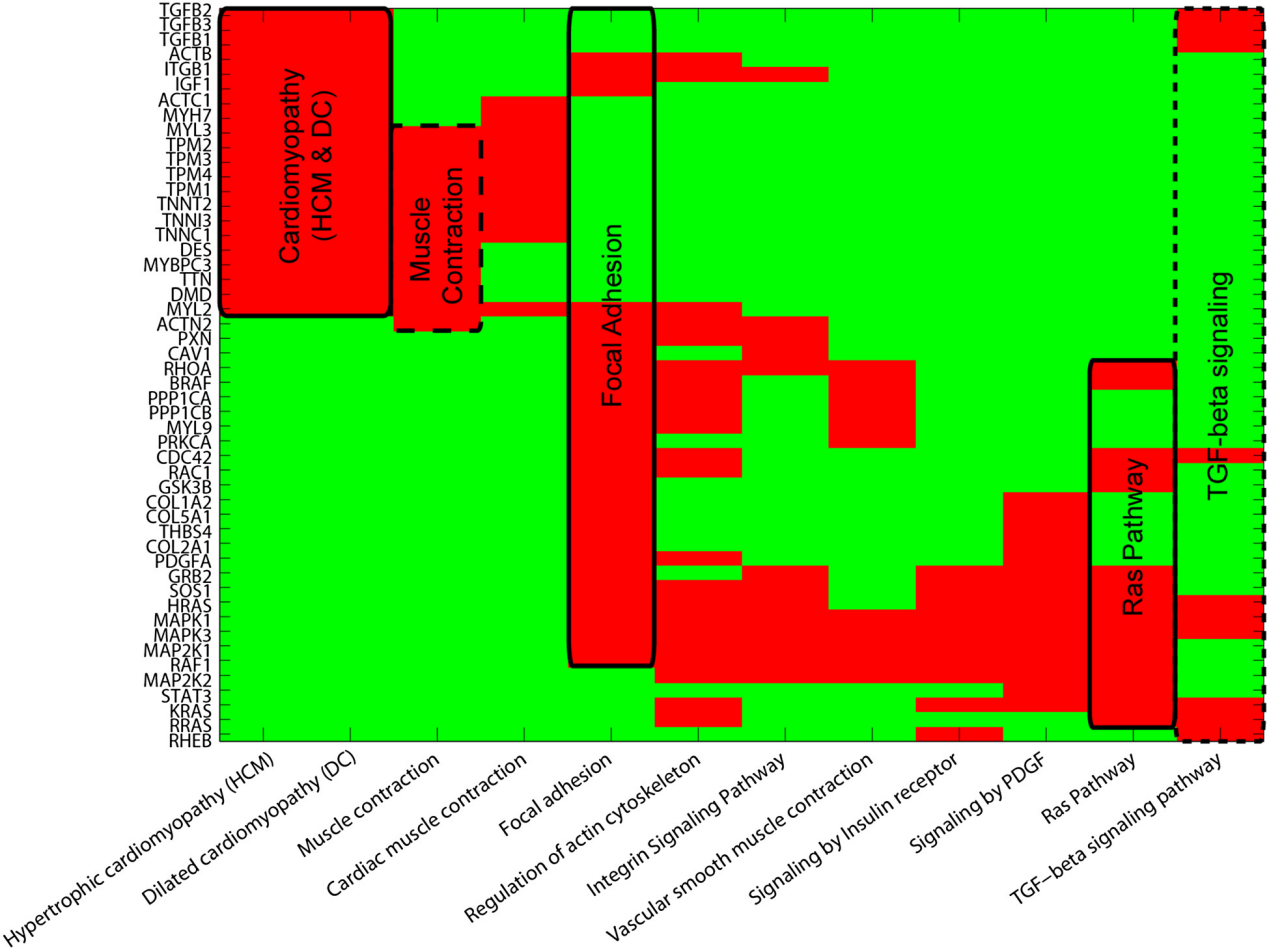
**Hierarchical bi-clustering of the CO candidate genes.** Hierarchical bi-clustering of the matrix of the CO candidate genes present in at least two pathways. The matrix consists of 50 candidate genes (rows) and 12 enriched pathways (columns). The black lines (full and dashed) are used to indicate the modules corresponding to clusters of interacting proteins in the respective pathways.

Hierarchical bi-clustering of the matrix [83], both in the rows and in the columns, was performed to detect clusters of genes which participated in common pathways, and clusters of pathways which share the same genes, respectively. The result of this analysis is provided in the Figure 4. The presence of two main groups of clusters is evident. The first group is constituted of “cardiomyopathy” (hypertrophic and dilated), “muscle contraction” and “cardiac muscle contraction” pathways. The second group is constituted of “focal adhesion”, “regulation of actin cytoskeleton, “integrin signaling”, “vascular smooth muscle contraction”, “signaling by insulin receptor”, “signaling by PDGF”, “RAS pathway”, and “TGF-beta signaling”.

**Figure 5:**
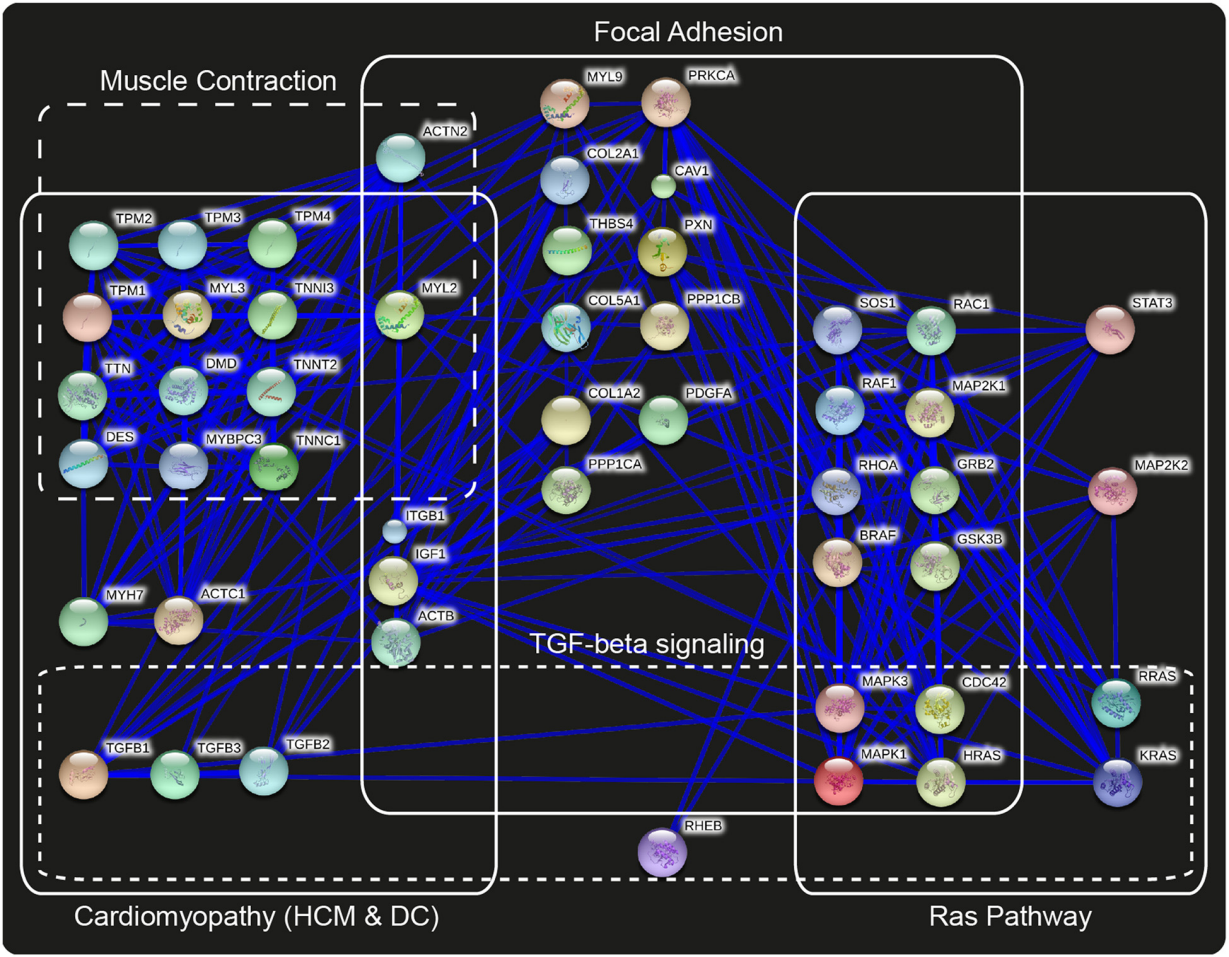
**String protein-protein interaction network (PPIN) of the CO candidate genes.** PPIN is obtained from the 50 CO candidate genes interacting in the STRING database. The lines (full and dashed) delimit the presence of the diverse overlapped protein-pathway-modules. The same line is used in the Figure 4 to indicate the modules corresponding to clusters of interacting proteins in the respective pathways.

In order to further investigate the relation between the genes involved in the “cardiomyopathy” (hypertrophic and dilated), the “muscle contraction” and the “RAS pathway” and to interpret their role in creating connections between the diverse pathway modules, we searched the STRING database [20] for protein-protein interactions, selecting only the interactions with high confidence score. The outcome of this analysis is represented in Figure 5. All the 50 genes presented at least one interaction in the PPI network produced by the STRING database. This network is provided as a supplementary material (Additional file 9: Table S9).

The principal pathways involved in both, CO and RASopathies, are displayed on the PPIN (Figure 5), and also marked in the Figure 4 to facilitate the comparison. This figure addresses the question of the relation between the common genetic mechanisms underlying CO and RASopathies. Figure 5 provides a clear visualization of the overlapping pathways and of the integrated network of relations existing on proteomic level. At the best of our knowledge, this is the first time that such relation is presented, and it might help in understanding the relation between co-presence of CO and cardiomyopathy as clinical and apparently unrelated features in RASopathies. This fact is clarified by the layout offered in Figure 5 that reveals how the “cardiomyopathy” and the “RAS signaling” pathways are connected by a plethora of interactions with high confidence score in the STRING database. To investigate the precise type of intra- and inter- pathway interactions we suggest to mine the network that we provide in the supplementary material (Additional file 9: Table S9). Figure 5 further emphasizes how the “focal adhesion” and the “TGF-beta signaling” pathways overlap the “cardiomyopathy”, the “muscle contraction” and the “RAS signaling” pathways by connecting proteins at different metabolic levels. The relevance of the “focal adhesion” pathway, as well as the importance of “cytoskeleton”, “muscle development”, “muscle contraction”, and “insulin signaling” pathways in cryptorchidism were widely discussed [7]. However, the referred study was conducted on a rat model and all of the pathways were considered and treated separately. Here, for the first time, we proceed to an integratomic investigation of the genetic factors linked to CO in human. Meanwhile, we offer the holistic perspective that points out how clinical features apparently unrelated with CO might be generated by genetic mutation(s) which propagate at different pathway levels of the network. This propagation on different pathway-modules can justify the onset of multiple unrelated clinical features in complex diseases, such as RASopathies. The selection of 43 network-based predicted genes considered together with these last disease-related evidences are another proof that confirms the power of PPIN for association of genes with diseases [21,84].

Our results are in concordance with previous observations that alignment of human interactome with human phenome enables identification of causative genes (and networks) underlying disease families. Phenotypic overlap implies genetic overlap and human phenome can be viewed as a landscape of interrelated diseases, which reflects overlapping molecular causation [6,85-88]. In addition, it has been already shown that causative genes from syndromes that are phenotypically similar to a genetically uncharacterized syndrome can be used to query the gene network for functionally related candidate genes [89].

### Candidate gene prioritization

Prioritization of candidate genes underlying complex traits remains one of the main challenges in molecular biology [11]. In this study we used three criteria for selecting the most promising candidate genes: 1) number of independent literature reports connecting the candidate gene with CO (Table 2), 2) involvement of candidate genes in enriched pathways (Table 3), and 3) position of candidate genes on the genomic map (genes positioned in regions where multiple CO associated data overlap were considered positional candidates) (Figure 2).

Twenty genes have been suggested as a genetic cause for CO in at least two independent studies (criterion 1) using different study approaches (*AMH*, *AMHR2*, *AR*, *ARID5B*, *BMP7*, *EPHA4*, *ESR1*, *FGFR2*, *HOXA10*, *HRAS*, *INSL3*, *LHCGR*, *MAP2K1*, *MSX1*, *NR5A1*, *RXFP2*, *SOS1*, *TNNI2*, *TNNT3*, and *WT1*). Among them, *INSL3* has been associated with CO in eight, *RXFP2* in five, and *AR* in four independent studies. However, this approach should be treated with some caution because of the possible bias towards research interest into more “popular” genes. The approach will be more reliable after significant amount of unbiased genome-wide studies is available.

Considering involvement in enriched pathways (criterion 2), the most promising candidates would be *HRAS*, *MAP2K1*, *MAP2K2*, *GRB2*, *RAF1* and *SOS1*, which are all involved in seven or more enriched pathways. For the literature-collected candidate genes involved in multiple (four or more) CO-enriched pathways we assembled genetic information relevant for further functional analyses: assignment to corresponding biological pathways, genetic variability, and putative presence of polymorphic microRNA (miRNA) target sites (Additional file 6: Table S6)*.* The importance of small non-coding RNAs (ncRNAs) in gene regulation and pathogenesis of the diseases, including reduced fertility, is today evident [90]. However, to our knowledge, there are no literature reports associating ncRNAs or epigenetic factors with CO.

The most promising candidates meeting both suggested criteria (1 and 2) are *FGFR2* (reported in two CO-associated studies/ involved in one CO-associated pathway), *HRAS* (3/8), *MAP2K1* (2/9), and *SOS1* (2/5). Additionally, *TNNI2* and *TNNT3* are reported in the literature (once each), involved in one CO enriched pathway (*i.e.* “muscle contraction”), and positioned in a region overlapping chromosomal mutation.

Genomic regions involved in the chromosome mutations on chromosomes 2, 4, 8, 9, 11, and X overlapped with 14 candidate genes suggested as positional candidates (criterion 3): *CAPG*, *MSX1*, *E2F5*, *PTCH1*, *BICD2*, *RPS6*, *FGFR2, HRAS*, *PAX6*, *WT1*, *TNNI2*, *TNN3*, *FLNA*, and *MECP2*. Additionally, three network-predicted candidate genes, *FHL2*, *TMOD1* and *MYBPC3* overlapped with chromosomal mutations. Considering suggested prioritization criteria, *HRAS* gene meets all of them.

Reliability of such methodologically different approaches is not always comparable (for example, data from genome-wide expression experiments is much less validated than syndromic or transgenic data); therefore, ranking candidate genes based only on a number of different reports/approaches is not always feasible. However, less validated data may also be of high biological relevance and should not be discarded for hypothesis-driven approaches. To increase reliability of the collected heterogeneous data we tested *in silico* how candidate genes interact at the proteomic level. Although integratomic approaches are only partially established yet and have several drawbacks, including already mentioned heterogeneity of input data, we believe that such approaches are a reasonable and at the moment among the most promising ways for hypothesis generation, which should be further experimentally validated in animal and/or human populations. Similar integratomics approach was already used for identification of candidate loci for mammary gland associated phenotypes [8], male infertility [9], and obesity [10,91], and could be adapted to any other complex trait.

## Conclusions

In this study we present an overview of CO associated candidate regions/genes and suggest pathways potentially involved in the pathogenesis of the disease. The integrative, comparative-genomics approach, and *in silico* analyses of the collected data aim to help solving the problem of fragmented and often contradictory data extracted from different methodologically focused studies. The protein-protein interactions analysis revealed the most relevant pathways associated with CO candidate gene list and enabled us to suggest additional candidate genes based on network prediction. Described systems biology approach will contribute to a better understanding of genetic causes for cryptorchidism and provides possible example how integration and linking of complex traits related data can be used for hypothesis generation. Publicly available online CO gene atlas and data entry option will allow researcher to enter, browse, and visualize CO associated data. The proposed network-based approach elucidates co-presence of similar pathogenetic mechanisms underlying diverse clinical syndromes/defects and could be of a great importance in research in the field of molecular syndromology. This approach has also a potential to be used for future development of diagnostic, prognostic, and therapeutic markers. The developed integratomics approach can be extrapolated to study genetic background of any other complex traits/diseases and to generate hypothesis for downstream experimental validation.

## Competing interests

The authors declare that they have no competing interests.

## Authors’ contributions

CVC performed pathway and PPIN analyses. JO, TK, and MZ performed the data mining and established the database. TK, JO, and CVC interpreted the results and drafted the manuscript. MZ developed web-based interactive visualization tool. PD and TR provided feedback on the initial draft and contributed to the final editing of the manuscript. All authors have read and approved the final manuscript.

## Pre-publication history

The pre-publication history for this paper can be accessed here:

http://www.biomedcentral.com/1755-8794/6/5/prepub

## Supplementary Material

Additional file 1: Table S1 Chromosomal abnormalities and CNVs associated with cryptorchidism. Click here for file

Additional file 2: Table S2 Selected clinical syndromes that feature CO in their clinical picture. Click here for file

Additional file 3: Table S3 Transgenic and knock-out murine models that display cryptorchid phenotype. Click here for file

Additional file 4: Table S4 Genes tested for association with CO. Click here for file

Additional file 5: Table S5 Genes with expression patterns associated with CO. Genes with expression patterns associated with CO in rat (adapted from [7]) and genomic location of their human orthologs. Click here for file

Additional file 6: Table S6 The literature collected candidate genes involved in multiple (four or more) CO-associated pathways. Click here for file

Additional file 7: Table S7 Forty-three network-predicted CO-associated candidate genes. Click here for file

Additional file 8: Table S8 Protein network information matrix for candidate genes. Protein network information matrix for candidate genes involved in at least two pathways significant for literature-collected and network-predicted candidate genes. The matrix consists of 50 rows (each row corresponds to a different gene involved in at least two pathways) and 12 columns (each column corresponds to a different pathway), where 0 indicates that the gene is not present in a pathway and 1 indicates that the gene is present in it. Click here for file

Additional file 9: Table S9 STRING network data: list of protein network interactions present in STRING with high confidence score. Click here for file
