# Supplementary material for: Pivotal role of the muscle-contraction pathway in cryptorchidism and evidence for genomic connections with cardiomyopathy pathways in RASopathies
Source: BMC Med Genomics. 2013 Feb 14;6:5. doi: 10.1186/1755-8794-6-5 (PMC3626861; doi:10.1186/1755-8794-6-5)
Supplement: Additional file 1: Table S1 — Chromosomal abnormalities and CNVs associated with cryptorchidism. [file 1755-8794-6-5-S1.doc]

| **Karyotype** | **Species** | **No. of cases** | **Reference** |
| --- | --- | --- | --- |
| ***Aneuploidies*** | | | |
| 47,XY,+r(18)(q10q11.2) | human | 1 |  |
| 45,X, 46,X,dic(Y), 47,X,dic(Y),dic(Y) | human |  |  |
| 47,XXY, 46,XY | human |  |  |
| 47,XXY, 46,XY, 46,XX | human |  |  |
| 47,XXY | human |  |  |
| 47,XYY | human |  |  |
| 47,XY + mar* | human | 2 |  |
| 47,XY, 10q- | human |  |  |
| 47,XY,+18 | human |  |  |
| 47,XXY, del (4)(p11pter) | human |  |  |
| 48,XXYY | human |  |  |
| 69,XXY | human |  |  |
| 59, XY,t(1;26) | cattle | 1 |  |
| 65, XY + 27 | horse | 1 |  |
| 78,XY/79,XXY | dog | 1 |  |
| ***Structural abnormalities*** | | | |
| 46,XX (sex reversal) | human |  |  |
| 46,XY,inv(Y)(p11q11) | human |  |  |
| 46,XY,inv(2)(p11q13) | human |  |  |
| 46, XY,inv(9)(p11q13) | human | 3 |  |
| 46,XY,t(8;9)(q21;q23) | human |  |  |
| 46,XY,t(10;11)(p13;p11) | human |  |  |
| 46,XY,t(1;2)(q25;q22) | human |  |  |
| 46,XY,t(2;4)(q21;p12) | human |  |  |
| 46,XX,t(9;11)(p22;p15.5) | human |  |  |
| 46,XY,der(22)t(22;Y) | human |  |  |
| 46,XY,dup(4)(p11p16) | human |  |  |
| 46, XY, del (9) (p23) | human | 1 |  |
| 46,XY, del (11)(p12p13) | human |  |  |
| 46,XY, del (10) (p14) | human | 1 |  |
| 46,XY, del (10) (q26) | human |  | OMIM |
| 46, XY, del (12) (q-subtelomeric region) | human | 1 |  |
| ***Copy number variants*** | | | |
| 46, XY, dup(10)(p14) | human | 1 |  |
| 46, XY, dup(X)(q28) | human | 1 |  |

*, marker chromosome.
