# Supplementary material for: Pivotal role of the muscle-contraction pathway in cryptorchidism and evidence for genomic connections with cardiomyopathy pathways in RASopathies
Source: BMC Med Genomics. 2013 Feb 14;6:5. doi: 10.1186/1755-8794-6-5 (PMC3626861; doi:10.1186/1755-8794-6-5)
Supplement: Additional file 2: Table S2 — Selected clinical syndromes that feature CO in their clinical picture. [file 1755-8794-6-5-S2.doc]

| **Syndrome** | **OMIM**  **(syndrome)** | **Gene** | **Location**  **(human)** | **Gene name** |
| --- | --- | --- | --- | --- |
| Beckwith-Wiedemann | 130650 | *NSD1* | 5q35 | nuclear receptor binding SET domain protein 1 |
|  |  | *CDKN1C* | 11p15.5 | cyclin-dependent kinase inhibitor 1C (p57, Kip2) |
| Aarskog-Scott | 100050 | *FGD1* | Xp11.21 | FYVE, RhoGEF and PH domain containing 1 |
| Testicular feminization | 300068 | *AR* | Xq11-q12 | androgen receptor |
| Opitz-Kaveggia (FG syndrome) | 305450 | *MED12 (FGS1)* | Xq12-q21.31 | FG syndrome 1 |
| Ichthyosis | 308100 | *STS* | Xp22.32 | steroid sulfatase (microsomal), isozyme S |
| Lowe oculocerebrorenal | 309000 | *OCRL* | Xq26.1 | oculocerebrorenal syndrome of Lowe |
| Gorlin – fronto-metaphyseal dysplasia | 305620 | *FLNA* | Xq28 | filamin A, alpha (actin binding protein 280) |
| Kallman | various types | *KAL1* | Xp22.3 | Kallmann syndrome 1 sequence |
|  |  | *PROKR2* | 20p12.3 | prokineticin receptor 2 |
|  |  | *PROK2* | 3p13 | Prokinectin 2 |
| Noonan | various types | *PTPN11* | 12q24.13 | protein tyrosine phosphatase, non-receptor type 11 |
|  |  | *SOS1* | 2p22-p21 | son of sevenless homolog 1 |
|  |  | *RAF1* | 3p25 | v-raf-1 murine leukemia viral oncogene homolog 1 |
|  |  | *KRAS* | 12p12.1 | v-Ki-ras2 Kirsten rat sarcoma viral oncogene homolog |
| Fanconi anemia (complementation groups E and O) | 600901 | *FANCE* | 6p22-p21 | Fanconi anemia, complementation group E |
|  | 613390 | *RAD51C* | 17q25.1 | DNA repair protein RAD51 homolog 3 |
| Amelogenesis imperfecta – polycistic renal dissease –cl/p | / | *MSX2* | 5q34-q35 | msh homeobox 2 |
| Sotos,  Weaver,  Beckwith-Weidemann | 117550  277590  130650 | *NSD1* | 5q35.2-q35.3 | nuclear receptor binding SET domain protein 1 |
| Apert – aerocephalosyndactyly type 1 | 101200 | *FGFR2* | 10q26 | fibroblast growth factor receptor 2 |
| Genitourinary dysplasia component of WAGR | 194072 | *WT1* | 11p13 | Wilms tumor 1 |
|  |  | *PAX6* | 11p13 | paired box 6 |
| Costello | 218040 | *HRAS* | 11p15.5 | v-Ha-ras Harvey rat sarcoma viral oncogene homolog |
| Distal arthrogryposis type 2B | 601680 | *TNNI2* | 11p15.5 | troponin I type 2 (skeletal, fast) |
|  |  | *TNNT3* | 11p15.5 | troponin T type 3 (skeletal, fast) |
| Cardiofaciocutaneous | 115150 | *KRAS* | 12p12.1 | v-Ki-ras2 Kirsten rat sarcoma viral oncogene homolog |
|  |  | *MAP2K1* | 15q22.1-q22.33 | mitogen-activated protein kinase kinase 1 |
|  |  | *MAP2K2* | 19p13.3 | mitogen-activated protein kinase kinase 2 |
|  |  | *BRAF* | 7q34 | v-raf murine sarcoma viral oncogene homolog B1 |
| Prader-Willi | 176270 | *SNRPN* | 15q11.2 | small nuclear ribonucleoprotein polypeptide N |
|  |  | *NDN* | 15q11.2-q12 | necdin homolog (mouse) |
| Distal arthrogryposis type 2A | 193700 | *MYH3* | 17p13.1 | myosin, heavy chain 3, skeletal muscle, embryonic |
| Prune Belly | 100100 | *CHRM3* | 1q43 | acetylcholine receptor, muscarinic, 3 |
| Micropthalmia, syndromic 3 | 206900 | *SOX2* | 3q26.33 | sry-related hmg-box gene 2 |
| Chromosome 10q26 deletion | 609625 | */* | 10q26 | / |
| Mental retardation, autosomal dominant 17 | 610443 | *KANSL1* | 17q21.31 | kat8 regulatory nsl complex subunit 1 |
| CHARGE | 214800 | *SEMA3E* | 7q21.11 | semaphorin 3e |
|  |  | *CHD7* | 8q12.1-q12.2 | chromodomain helicase dna-binding protein 7 |
| Leopard 1 | 151100 | *PTPN11* | 12q24.13 | protein-tyrosine phosphatase, nonreceptor-type, 11 |
| Popliteal pterygium | 119500 | *IRF6* | 1q32.2 | interferon regulatory factor 6 |
| **Leydig** **cell** hypoplasia, type I | 238320 | *LHCGR* | 2p16.3 | luteinizing hormone/choriogonadotropin receptor |
| IFAP/BRESHECK | 308205 | *MBTPS2* | Xp22.12-p22.11 | membrane-bound transcription factor protease, site 2 |
| Corpus callosum, agenesis of, with abnormal genitalia | 300004 | *ARX* | Xp21.3 | aristaless-related homeobox, x-linked |
| Mental retardation-hypotonic facies syndrome, x-linked | 309580 | *ATRX* | Xq21.1 | alpha thalassemia/mental retardation syndrome X-linked |
| Chromosome 10q26 deletion | 609625 | */* | 10q26 | / |
| Persistent mullerian duct, types I and II | 261550 | *AMHR2* | 12q13.13 | anti-mullerian hormone type II receptor |
|  |  | *AMH* | 19p13.13 | anti-mullerian hormone |
