# Supplementary material for: Pivotal role of the muscle-contraction pathway in cryptorchidism and evidence for genomic connections with cardiomyopathy pathways in RASopathies
Source: BMC Med Genomics. 2013 Feb 14;6:5. doi: 10.1186/1755-8794-6-5 (PMC3626861; doi:10.1186/1755-8794-6-5)
Supplement: Additional file 3: Table S3 — Transgenic and knock-out murine models that display cryptorchid phenotype. [file 1755-8794-6-5-S3.doc]

| **Gene** | **Chr.** | **Location** | **Location (human)** | **Gene name** | **Reference** |
| --- | --- | --- | --- | --- | --- |
| ***Mouse models*** | | | | | |
| *Epha4* | 1 | 43 cM | 2q36.1 | Eph receptor A4 | MGI |
| *Lbr* | 1 | 97.3 cM | 1q42.1 | lamin B receptor | MGI |
| *Dnajc5* | 2 | 106 cM | / | DnaJ (Hsp40) homolog, subfamily C, member 5 | MGI |
| *Lrp2* | 2 | 40 cM | 2q24-q31 | low density lipoprotein receptor-related protein 2 | MGI |
| *Nr5a1* | 2 | 24 | 9q33 | nuclear receptor subfamily 5, group A, member 1 | MGI |
| *Scg5* | 2 | 64 cM | 15q13-q14 | secretogranin V | MGI |
| *Wt1* | 2 | 58.0 cM | 11 (p13) | Wilms tumor 1 homolog |  |
| *Nhlh2* | 3 | 01814078-101818429 bp | 1p12-p11 | nescient helix loop helix 2 | MGI |
| *Crsp* | 5 | 84 cM | / | CO with white spotting, deletion region | MGI |
| *Gnrhr* | 5 | 44 cM | 4q21.2 | gonadotropin releasing hormone receptor | MGI |
| *Rxfp2* | 5 | 84 cM | 13q13.1 | relaxin/insulin-like family peptide receptor 2 | MGI |
| *Fkbp4* | 6 | 128379753-128388695 bp | 12p13.33 | FK506 binding protein 4 | MGI |
| *Hoxa10* | 6 | 26.33 cM | 7p15-p14 | homeo box A10 | MGI |
| *Hoxa11* | 6 | 26.33 cM | 7p15-p14 | homeo box A11 | MGI |
| *Ret* | 6 | 53.2 cM | 10q11.2 | ret proto-oncogene | MGI |
| *Hmgb2* | 8 | 31 cM | 4q31 | high mobility group box 2 | MGI |
| *Insl3* | 8 | 33 cM | 19p13.2-p12 | insulin-like 3 | MGI |
| *Cyp19a1* | 9 | 31 cM | 15 (q21.1) | cytochrome P450, family 19, subfamily a, polypeptide 1 |  |
| *Bmp5* | 9 | 42 cM | 6p12.1 | bone morphogenetic protein 5 | MGI |
| *Arid5b* | 10 | 10 cM | 10q21.2 | AT rich interactive domain 5B (Mrf1 like) | MGI |
| *Amh* | 10 | 33 cM | 19p13.3 | anti-Mullerian hormone | MGI |
| *Hmga2* | 10 | 68cM | 12q15 | high mobility group AT-hook 2 | MGI |
| *Esr1* | 10 | 12 cM | 6q25.1 | estrogen receptor 1 |  |
| *Nog* | 11 | 54cM | 17q22 | noggin | MGI |
| *Sox9* | 11 | 77 cM | 17q23 | SRY-box containing gene 9 | MGI |
| *Ptch1* | 13 | 33 cM | 9q22.3 | patched homolog 1 | MGI |
| *Gnrh1* | 14 | 30,5 cM | 8p21-p11.2 | gonadotropin releasing hormone 1 | MGI |
| *Dhh* | 15 | 57.4 cM | 12q12-q13.1 | desert hedgehog | MGI |
| *Amhr2* | 15 | 57.58 | 12 | anti-Mullerian hormone type 2 receptor | MGI |
| *Parl* | 16 | 14 cM | 3q27.1 | presenilin associated, rhomboid-like | MGI |
| *Lhcgr* | 17 | 46.5 cM | 2p21 | luteinizing hormone/choriogonadotropin receptor | MGI |
| *Sox8* | 17 | 8 cM | 16p13.3 | SRY-box containing gene 8 | MGI |
| *H2* | 17 | UN | / | histocompatibility-2, MHC | MGI |
| *Ar* | X | 36 cM | Xq11.2-q12 | androgen receptor | MGI |
| *Foxp3* | X | 2.1 cM | Xp11.23 | forkhead box P3 | MGI |
| *Mecp2* | X | 29.6 cM | Xq28 | methyl CpG binding protein 2 | MGI |
| *Nr0b1* | X | 33.0 cM | Xp21.3-p21.2 | nuclear receptor subfamily 0, group B, member 1 |  |
| *Xpl* | X | UN | / | X-linked polydactyly | MGI |
| *Ww1* | UN | UN | / | small papilla 1 | MGI |
| ***Rat model*** | | | | | |
| *Crcp (Cgrp-rcp)* | 12 | q13 | 7q11.21 | CGRP receptor component |  |
