# Supplementary material for: Pivotal role of the muscle-contraction pathway in cryptorchidism and evidence for genomic connections with cardiomyopathy pathways in RASopathies
Source: BMC Med Genomics. 2013 Feb 14;6:5. doi: 10.1186/1755-8794-6-5 (PMC3626861; doi:10.1186/1755-8794-6-5)
Supplement: Additional file 4: Table S4 — Genes tested for association with CO. [file 1755-8794-6-5-S4.doc]

| **Gene** | **Species** | **Location (human)** | **Gene name** | **Reference** |
| --- | --- | --- | --- | --- |
| *TGFBR3** | human | 1p33-p32 | transforming growth factor, beta receptor III | (+) |
| *BMP7** | human | 20q13 | bone morphogenetic protein 7 | (+) |
| *Esr1* | human | 6q25.1 | estrogen receptor 1 | (+) |
|  |  |  |  | (+) |
|  |  |  |  | (+) |
|  |  |  |  | (-) |
|  |  |  |  | (-) |
|  |  |  |  | (-) |
| *NR5A1* (*SF-1*) | human | 9q33 | nuclear receptor subfamily 5, group A, member 1 | (+) |
| *HOXA10* | human | 7p15.2 | homeobox A10 | (+) |
|  |  |  |  | (-) |
| *Rxfp2* (*LGR8/*  *great*) | human | 13q13.1 | relaxin/insulin-like family peptide receptor 2 | (+) |
|  |  |  |  | (+) |
|  |  |  |  | (+) |
|  |  |  |  | (+) |
|  |  |  |  | (-) |
| *Insl3* | human | 19p13.2-p12 | insulin-like 3 (Leydig cell) | (+) |
|  |  |  |  | (+) |
|  |  |  |  | (+) |
|  |  |  |  | (+) |
|  |  |  |  | (+) |
|  |  |  |  | (-) |
|  |  |  |  | (-) |
|  |  |  |  | (-) |
| *AR* | human | Xq11.2-q12 | androgen receptor | (+) |
|  |  |  |  | (+) |
| *Kiss1R* | human | 19p13.3 | KISS1 receptor | (+) |
| *Lhcgr* | human | 2p21 | luteinizing hormone/choriogonadotropin receptor | (-) |
| *AZF1* | human | Yq | Azoospermia factor 1 | (-) |
|  |  |  |  | (-) |
|  |  |  |  | (-) |
| *CALCA (CGRP)* | human | 11p15.2 | calcitonin-related polypeptide alpha | (-) |
| *Insl3* | sheep | ND | insulin-like 3 (Leydig cell) | (+) |
| *COL2A1* | dog | 12q13.11 | collagen, type II, alpha 1 | (+) |
| *INSL3* | dog | 20 | insulin-like 3 (Leydig cell) | (+) |

ND, not defined, +, positive association; -, no association, *, combination of GWAS and integration of other data.
