# Supplementary material for: Pivotal role of the muscle-contraction pathway in cryptorchidism and evidence for genomic connections with cardiomyopathy pathways in RASopathies
Source: BMC Med Genomics. 2013 Feb 14;6:5. doi: 10.1186/1755-8794-6-5 (PMC3626861; doi:10.1186/1755-8794-6-5)
Supplement: Additional file 5: Table S5 — Genes with expression patterns associated with CO. Genes with expression patterns associated with CO in rat (adapted from [7]) and genomic location of their human orthologs. [file 1755-8794-6-5-S5.doc]

| **Gene** | **Chromosome location (human)** | **Gene name** |
| --- | --- | --- |
| *Myog* | 1q31-q41 | myogenin |
| *Tnnt2* | 1q32 | troponin T type 2 (cardiac) |
| *Igfbp5* | 2q33-q36 | insulin-like growth factor binding protein 5 |
| *Msx1* | 4p16.3-16.1 | mshhomeobox 1 |
| *Fst* | 5q11.2 | follistatin |
| *Id2* | 6q16 | inhibitor of DNA binding 2 |
| *Igf1* | 12q22-q23 | insulin-like growth factor 1 (somatomedin C) |
| *Jag1* | 20p12.1-p11.23 | jagged 1 |
| *Bmp4* | 14q22-q23 | bone morphogenetic protein 4 |
| *SIX1* | 14q23.1 | SIX homeobox 1 |
| *igfbp3* | 7p13-p12 | insulin-like growth factor binding protein 3 |
| *TTN* | 2q31 | titin |
| *rb1* | 13q14.2 | retinoblastoma 1 |
| *myh7* | 14q12 | myosin, heavy chain 7, cardiac muscle, beta |
| *myf6* | 12q21 | myogenic factor 6 (herculin) |
| *tagln2* | 1q21-q25 | transgelin 2 |
| *des* | 2q35 | desmin |
| *acta1* | 1q42.13 | actin, alpha 1, skeletal muscle |
| *fhl3* | 1p34 | four and a half LIM domains 3 |
| *capn3* | 15q15.1-q21.1 | calpain 3 |
| *tagln* | 11q23.2 | transgelin |
| *myl9* | 20q11.23 | myosin, light chain 9, regulatory |
| *LGALS1* | 22q13.1 | lectin, galactoside-binding, soluble |
| *ALdoa* | 16p11.2 | aldolase A, fructose-bisphosphate |
| *acta2* | 10q23.3 | actin, alpha 2, smooth muscle, aorta |
| *MYL3* | 3p21.3-p21.2 | myosin, light chain 3, alkali; ventricular, skeletal, slow |
| *trdn* | 6q22-q23 | triadin |
| *tpm4* | 19p13.1 | tropomyosin 4 |
| *tpm1* | 1q21.2 | tropomyosin 1 |
| *tpm3* | 15q22.1 | tropomyosin 3 |
| *myl2* | 12q24.11 | myosin, light chain 2, regulatory, cardiac, slow |
| *fat1* | 4q35 | FAT tumor suppressor homolog 1 |
| *SMC3* | 10q25 | structural maintenance of chromosomes 3 |
| *tnk2* | 3q29 | tyrosine kinase, non-receptor, 2 |
| *EZR* | 6q25.2-q26 | ezrin |
| *wipF1* | 2q31.1 | WAS/WASL interacting protein family, member 1 |
| *bicd2* | 9q22.31 | bicaudal D homolog 2 (Drosophila) |
| *cap2* | 6p22.3 | CAP, adenylatecyclase-associated protein, 2 (yeast) |
| *tbcb* | 19q13.11-q13.12 | tubulin folding cofactor B |
| *dstn* | 20p12.1 | destrin (actin depolymerizing factor) |
| *pfn1* | 17p13.3 | profilin 1 |
| *bin3* | 8p21.3 | bridging integrator 3 |
| *pgls* | 19p13.2 | 6-phosphogluconolactonase |
| *capg* | 2p11.2 | capping protein (actin filament), gelsolin-like |
| *cfl1* | 11q13 | cofilin 1 (non-muscle) |
| *dctn3* | 9p13 | dynactin 3 (p22) |
| *abr* | 17p13.3 | active BCR-related gene |
| *rab18* | 10p12.1 | RAB18, member RAS oncogene family |
| *cdc42* | 1p36.1 | cell division cycle 42 (GTP binding protein, 25kDa) |
| *farp2* | 2q37.3 | FERM, RhoGEF and pleckstrin domain protein 2 |
| *rab2b* | 14q11.2 | RAB2B, member RAS oncogene family |
| *cdc42ep4* | 17q24-q25 | CDC42 effector protein (Rho GTPase binding) 4 |
| *ralbp1* | 18p11.3 | ralA binding protein 1 |
| *sos1* | 2p22-p21 | son of sevenless homolog 1 (Drosophila) |
| *hras* | 11p15.5 | v-Ha-ras Harvey rat sarcoma viral oncogene homolog |
| *rab7l1* | 1q32 | RAB7, member RAS oncogene family-like 1 |
| *rnd2* | 17q21 | Rho family GTPase 2 |
| *rac1* | 7p22 | ras-related C3 botulinum toxin substrate 1 (rho family, small GTP binding protein Rac1) |
| *rab7A* | 3q21.3 | RAB7A, member RAS oncogene family |
| *arhgap32* | 11q24-q25 | Rho GTPase activating protein 32 |
| *arhgdip* | 12p12.3 | Rho GDP dissociation inhibitor (GDI) beta |
| *ywhaq* | 2p25.1 | tyrosine 3-monooxygenase/tryptophan 5-monooxygenase activation protein, theta polypeptide |
| *synj2bp* | 14q24.2 | synaptojanin 2 binding protein |
| *rhoa* | 3p21.3 | ras homolog gene family, member A |
| *rras* | 19q13.3-qter | related RAS viral (r-ras) oncogene homolog |
| *wnt4* | 1p36.23-p35.1 | wingless-type MMTV integration site family, member 4 |
| *dusp6* | 12q22-q23 | dual specificity phosphatase 6 |
| *olfm1* | 9q34.3 | olfactomedin 1 |
| *kitl* | 12q22 | KIT ligand |
| *wisp1* | 8q24.1-q24.3 | WNT1 inducible signaling pathway protein 1 |
| *sfrp2* | 4q31.3 | secreted frizzled-related protein 2 |
| *rarb* | 3p24 | retinoic acid receptor, beta |
| *fgf9* | 13q11-q12 | fibroblast growth factor 9 (glia-activating factor) |
| *fgfr1* | 8p11.2-p11.1 | fibroblast growth factor receptor 1 |
| *nfkb1* | 4q24 | nuclear factor of kappa light polypeptide gene enhancer in B-cells 1 |
| *fgfr2* | 10q26 | fibroblast growth factor receptor 2 |
| *gpc3* | Xq26.1 | glypican 3 |
| *stat3* | 17q21.31 | signal transducer and activator of transcription 3 (acute-phase response factor) |
| *FOS* | 14q24.3 | FBJ murine osteosarcoma viral oncogene homolog |
| *Timp1* | Xp11.3-p11.23 | TIMP metallopeptidase inhibitor 1 |
| *e2f5* | 8q21.2 | transcription factor E2F5 |
| *bmp7* | 20q13 | bone morphogenetic protein 7 |
| *timp2* | 17q25 | TIMP metallopeptidase inhibitor 2 |
| *foxo1a* | 13q14.1 | forkhead box O1 |
| *ppp1cb* | 2p23 | protein phosphatase 1, catalytic subunit, beta isozyme |
| *eif4e* | 4q21-q25 | eukaryotic translation initiation factor 4E |
| *rps6* | 9p21 | ribosomal protein S6 |
| *rps6kb1* | 17q23.1 | ribosomal protein S6 kinase, 70kDa, polypeptide 1 |
| *eif4ebp1* | 8p12 | eukaryotic translation initiation factor 4E binding protein 1 |
| *col5a1* | 9q34.2-q34.3 | collagen, type V, alpha 1 |
| *gsk3b* | 3q13.3 | glycogen synthase kinase 3 beta |
| *vcf* | 22q11.21-q11.23 | velocardiofacial syndrome |
| *map2k1* | 15q22.1-q22.33 | mitogen-activated protein kinase kinase 1 |
| *pdgfa* | 7p22 | platelet-derived growth factor alpha polypeptide |
| *col1a2* | 7q22.1 | collagen, type I, alpha 2 |
| *pik3r4* | 3q22.1 | phosphoinositide-3-kinase, regulatory subunit 4 |
| *ccnd1* | 11q13 | cyclin D1 |
| *thbs4* | 5q13 | thrombospondin 4 |
| *pxn* | 12q24.31 | paxillin |
| *ilk* | 11p15.5-p15.4 | integrin-linked kinase |
| *ppp1ca* | 11q13 | protein phosphatase 1, catalytic subunit, alpha isozyme |
| *grb2* | 17q24-q25 | growth factor receptor-bound protein 2 |
| *Actb* | 7p15-p12 | actin, beta |
| *itgb1* | 10p11.2 | integrin, beta 1 (fibronectin receptor, beta polypeptide, antigen CD29 includes MDF2, MSK12) |
| *ephA4* | 2q36.1 | EPH receptor A4 |
| *arid5B* | 10q21.2 | AT rich interactive domain 5B (MRF1-like) |
| *RYR1* | 19q13.1 | ryanodine receptor 1 (skeletal) |
| *bbs2* | 16q21 | Bardet-Biedl syndrome 2 |
| *hoxa10* | 7p15-p14 | homeobox A10 |
