# Supplementary material for: Pivotal role of the muscle-contraction pathway in cryptorchidism and evidence for genomic connections with cardiomyopathy pathways in RASopathies
Source: BMC Med Genomics. 2013 Feb 14;6:5. doi: 10.1186/1755-8794-6-5 (PMC3626861; doi:10.1186/1755-8794-6-5)
Supplement: Additional file 6: Table S6 — The literature collected candidate genes involved in multiple (four or more) CO-associated pathways. [file 1755-8794-6-5-S6.docx]

| **Gene^1^** | **Corresponding pathways (DAVID)** | **Number of genetic variations (probably damaging)^2^** | **Polymorphic miRNA target sites^3^**  **SNP ID / (hsa-miR-)** |
| --- | --- | --- | --- |
| *ACTB* | focal adhesion, regulation of actin cytoskeleton, hypertrophic cardiomyopathy, dilated cardiomyopathy | 94 (17) | rs11266786;  rs3210647;  rs11546891 (1274a);  rs11546927 (486-5p);  rs11546941,  rs268571 |
| *GRB2* | focal adhesion, signaling by insulin receptor, signaling by PDGF, RAS pathway, signaling by EGFR, IGF-1 signaling, integrin signaling | 29 (5) | rs11294952 (626, 370);  rs11290288 (626, 370);  rs7219 (302b*, 302a*, 1288) |
| *CDC42* | regulation of actin cytoskeleton, focal adhesion, signaling by EGFR, RAS pathway, role of MAL in Rho-mediated activation of SRF, integrin signaling | 19 (1) | rs11544899 (1322, 1272),  rs11544897 (33a*),  rs34949201 ,  rs28463010 (520a-5p. 525a-5p),  rs1803270 (1200) |
| ***HRAS*** | regulation of actin cytoskeleton, focal adhesion, signaling by PDGF, signaling by insulin receptor, signaling by EGFR, RAS pathway, role of MAL in Rho-mediated activation of SRF, IGF-1 signaling, integrin signaling, | 110 (18) | rs45592334 (149*);  rs41303613 (149*) |
| *IGF1* | focal adhesion, IGF-1 signaling, hypertrophic cardiomyopathy, dilated cardiomyopathy | 28 (5) | rs35365615 (219-2-3p),  rs6218 (603),  rs6217,  rs17847188,  rs17886337 (30a* 30e*, 30d*),  rs17879349 (219-1-3p),  rs3730203,  rs3032449 (656),  rs5031032 (613, 1, 206),  rs14970  rs5742698 (668) |
| *ITGB1* | regulation of actin cytoskeleton, focal adhesion, hypertrophic cardiomyopathy, integrin signaling, dilated cardiomyopathy | 152 (31) | rs1049433 (30a, 30b, 30c, 30d, 30e, let-7f-2*);  rs11557913 (320a, 320b, 320c, 320d);  rs11009140 (296-3p);  rs35422295 (1208);  rs11009146 (607);  rs2230396 (1237) |
| *KRAS* | regulation of actin cytoskeleton, signaling by insulin receptor, signaling by PDGF, RAS pathway, signaling by EGFR, integrin signaling | 160 (60) | rs12245 (544);  rs1137188 (511);  rs4963858 (147, 147b);  rs1141948 (544);  rs7973450 (302c*);  rs9266 (181a, 181b, 181c, 181d);  rs1141947;  rs712 (877) |
| ***MAP2K1*** | regulation of actin cytoskeleton, focal adhesion, signaling by PDGF, signaling by insulin receptor, RAS pathway, signaling by EGFR, role of MAL in Rho-mediated activation of SRF, IGF-1 signaling, integrin signaling | 73 (11) | rs34321222 (920, 939) |
| *MAP2K2* | regulation of actin cytoskeleton, signaling by PDGF, signaling by insulin receptor, signaling by EGFR, RAS pathway, role of MAL in Rho-mediated activation of SRF, integrin signaling | 93 (12) | rs3189510 |
| *MYL2* | muscle contraction, focal adhesion, regulation of actin cytoskeleton, hypertrophic cardiomyopathy, dilated cardiomyopathy | 58 (11) | rs1803294 (1265) |
| *PXN* | regulation of actin cytoskeleton, focal adhesion, signaling by EGFR, integrin signaling | 59 (2) | [rs12423375](http://www.ncbi.nlm.nih.gov/SNP/snp_ref.cgi?rs=rs12423375) (768-5p, 149*, 30b*),  rs10128770 (1230) |
| *RAC1* | regulation of actin cytoskeleton, focal adhesion, RAS pathway, role of MAL in Rho-mediated activation of SRF, integrin signaling | 30 (1) | *rs12977* (423-3p),  *rs34625328* (298),  rs36059496,  rs35197465,  *rs14627* (382, let-7g*) |
| *RAF1* | regulation of actin cytoskeleton, focal adhesion, signaling by PDGF, signaling by insulin receptor, signaling by EGFR, RAS pathway, role of MAL in Rho-mediated activation of SRF, IGF-1 signaling, integrin signaling, | 115 (21) | rs5746251 (708*),  rs5746248 (1288),  rs3730298 (146a*) |
| *RHOA* | regulation of actin cytoskeleton, focal adhesion, RAS pathway, role of MAL in Rho-mediated activation of SRF, integrin signaling | 27 (7) | rs11552766 (185),  rs3204561 (146a, 146b-5p),  rs3191115 (30b*),  rs9609,  rs1804289 (603),  rs14566 (559, 548),  rs15932 (183) |
| ***SOS1*** | regulation of actin cytoskeleton, focal adhesion, signaling by PDGF, signaling by insulin receptor, signaling by EGFR, RAS pathway, IGF-1 signaling, integrin signaling | 186 (20) | [rs11124658](http://www.ncbi.nlm.nih.gov/SNP/snp_ref.cgi?rs=rs11124658) (199a-5p, 199b-5p);  [rs1011706](http://www.ncbi.nlm.nih.gov/SNP/snp_ref.cgi?rs=rs1011706) (548d-3p);  [rs1059313](http://www.ncbi.nlm.nih.gov/SNP/snp_ref.cgi?rs=rs1059313) (212, 132);  [rs35478027](http://www.ncbi.nlm.nih.gov/SNP/snp_ref.cgi?rs=rs35478027) (106a) |

^1^ , bold denotes genes reported in the literature by multiple (at least two) independent studies

^2^, predicted by PolyPhen in ENSEMBL protein variation information,

^3^, according to the Patrocles database, SNPs in italics validated by the 1000 Genomes Project (human) or with known frequencies (others)
