# Supplementary material for: Pivotal role of the muscle-contraction pathway in cryptorchidism and evidence for genomic connections with cardiomyopathy pathways in RASopathies
Source: BMC Med Genomics. 2013 Feb 14;6:5. doi: 10.1186/1755-8794-6-5 (PMC3626861; doi:10.1186/1755-8794-6-5)
Supplement: Additional file 7: Table S7 — Forty-three network-predicted CO-associated candidate genes. [file 1755-8794-6-5-S7.doc]

| **Symbol** | **Gene Name** | **p-value** |
| --- | --- | --- |
| *MYH8* | myosin, heavy chain 8, skeletal muscle, perinatal | 6,13E-08 |
| *MYL1* | myosin, light chain 1, alkali; skeletal, fast | 8,92E-08 |
| *TPM2* | tropomyosin 2 (beta) | 2,43E-07 |
| *MYBPC2* | myosin binding protein C, fast type | 3,00E-07 |
| *MSTN* | myostatin | 4,54E-07 |
| *SHH* | sonic hedgehog homolog (Drosophila) | 5,84E-07 |
| *LEFTY1* | left-right determination factor 1 | 1,11E-06 |
| *TGFBI* | transforming growth factor, beta-induced, 68kDa | 2,10E-06 |
| *TNNC2* | troponin C type 2 (fast) | 2,33E-06 |
| *MYL4* | myosin, light chain 4, alkali; atrial, embryonic | 3,65E-06 |
| *NODAL* | nodal homolog (mouse) | 4,72E-06 |
| *TNNC1* | troponin C type 1 (slow) | 8,00E-06 |
| *TMOD1* | tropomodulin 1 | 8,16E-06 |
| *NEB* | nebulin | 9,86E-06 |
| *TGFB1* | transforming growth factor, beta 1 | 1,08E-05 |
| *MYBPC1* | myosin binding protein C, slow type | 1,19E-05 |
| *LEFTY2* | left-right determination factor 2 | 1,56E-05 |
| *TNNI1* | troponin I type 1 (skeletal, slow) | 1,69E-05 |
| *TGFB2* | transforming growth factor, beta 2 | 1,89E-05 |
| *MYBPC3* | myosin binding protein C, cardiac | 2,91E-05 |
| *TCAP* | titin-cap (telethonin) | 2,99E-05 |
| *MAPK1* | mitogen-activated protein kinase 1 | 1,80E-04 |
| *CAV1* | caveolin 1, caveolae protein, 22kDa | 2,06E-04 |
| *TGFB3* | transforming growth factor, beta 3 | 2,29E-04 |
| *PRKCA* | protein kinase C, alpha | 3,88E-04 |
| *BMP2* | bone morphogenetic protein 2 | 5,07E-04 |
| *TNNT1* | troponin T type 1 (skeletal, slow) | 0,001001 |
| *MYH11* | myosin, heavy chain 11, smooth muscle | 0,001195 |
| *TNNI3* | troponin I type 3 (cardiac) | 0,001298 |
| *MAPK3* | hypothetical LOC100271831; mitogen-activated protein kinase 3 | 0,00159 |
| *ANGPT1* | angiopoietin 1 | 0,002253 |
| *VIM* | vimentin | 0,002722 |
| *FGF3* | fibroblast growth factor 3 (murine mammary tumor virus integration site (v-int-2) oncogene homolog) | 0,002991 |
| *FHL2* | four and a half LIM domains 2 | 0,00363 |
| *DMD* | dystrophin | 0,004071 |
| *ACTN2* | actinin, alpha 2 | 0,004072 |
| *PRKCZ* | protein kinase C, zeta | 0,005304 |
| *RHEB* | Ras homolog enriched in brain | 0,005688 |
| *IGF1R* | insulin-like growth factor 1 receptor | 0,005691 |
| *PRKCE* | protein kinase C, epsilon | 0,006049 |
| *RAP1GDS1* | RAP1, GTP-GDP dissociation stimulator 1 | 0,007242 |
| *ACTC1* | actin, alpha, cardiac muscle 1 | 0,008059 |
| *SHBG* | sex hormone-binding globulin | 0,009741 |
