# Supplementary material for: Pivotal role of the muscle-contraction pathway in cryptorchidism and evidence for genomic connections with cardiomyopathy pathways in RASopathies
Source: BMC Med Genomics. 2013 Feb 14;6:5. doi: 10.1186/1755-8794-6-5 (PMC3626861; doi:10.1186/1755-8794-6-5)
Supplement: Additional file 9: Table S9 — STRING network data: list of protein network interactions present in STRING with high confidence score. [file 1755-8794-6-5-S9.docx]

| **Node1** | **Node2** | **Neighborhood** | **Fusion** | **Cooccurence** | **Homology** | **Coexpression** | **Experimental** | **Knowledge** | **Textmining** | **Combined score** |
| --- | --- | --- | --- | --- | --- | --- | --- | --- | --- | --- |
| *SOS1* | *RAC1* | 0.000 | 0.000 | 0.000 | 0.000 | 0.000 | 0.000 | 0.900 | 0.711 | 0.969 |
| *KRAS* | *MAPK1* | 0.000 | 0.000 | 0.000 | 0.000 | 0.000 | 0.000 | 0.800 | 0.633 | 0.921 |
| *CDC42* | *PRKCA* | 0.000 | 0.000 | 0.000 | 0.000 | 0.000 | 0.531 | 0.000 | 0.953 | 0.976 |
| *MAPK3* | *MAP2K2* | 0.000 | 0.000 | 0.000 | 0.664 | 0.000 | 0.987 | 0.800 | 0.993 | 0.998 |
| *SOS1* | *HRAS* | 0.000 | 0.000 | 0.000 | 0.000 | 0.000 | 0.999 | 0.900 | 0.858 | 0.999 |
| *DES* | *ACTN2* | 0.000 | 0.000 | 0.000 | 0.000 | 0.000 | 0.000 | 0.900 | 0.578 | 0.955 |
| *SOS1* | *KRAS* | 0.000 | 0.000 | 0.000 | 0.000 | 0.000 | 0.000 | 0.900 | 0.955 | 0.995 |
| *COL2A1* | *TGFB1* | 0.000 | 0.000 | 0.000 | 0.000 | 0.000 | 0.620 | 0.000 | 0.943 | 0.977 |
| *SOS1* | *GRB2* | 0.000 | 0.000 | 0.000 | 0.000 | 0.000 | 0.999 | 0.900 | 0.990 | 0.999 |
| *MAP2K1* | *MAPK3* | 0.000 | 0.000 | 0.000 | 0.649 | 0.000 | 0.992 | 0.900 | 0.994 | 0.999 |
| *SOS1* | *MAPK3* | 0.000 | 0.000 | 0.000 | 0.000 | 0.000 | 0.000 | 0.900 | 0.638 | 0.961 |
| *PRKCA* | *MAPK3* | 0.000 | 0.000 | 0.000 | 0.641 | 0.000 | 0.000 | 0.900 | 0.781 | 0.927 |
| *MYH7* | *MYL9* | 0.000 | 0.000 | 0.000 | 0.000 | 0.000 | 0.000 | 0.900 | 0.765 | 0.974 |
| *HRAS* | *MAP2K2* | 0.000 | 0.000 | 0.000 | 0.000 | 0.000 | 0.000 | 0.000 | 0.798 | 0.798 |
| *TNNI3* | *TPM1* | 0.000 | 0.000 | 0.000 | 0.000 | 0.000 | 0.000 | 0.900 | 0.987 | 0.998 |
| *ACTN2* | *TGFB1* | 0.000 | 0.000 | 0.000 | 0.000 | 0.000 | 0.000 | 0.900 | 0.000 | 0.899 |
| *ACTB* | *TPM1* | 0.000 | 0.000 | 0.000 | 0.000 | 0.000 | 0.620 | 0.000 | 0.464 | 0.782 |
| *RAC1* | *CAV1* | 0.000 | 0.000 | 0.000 | 0.000 | 0.000 | 0.620 | 0.000 | 0.948 | 0.978 |
| *HRAS* | *PRKCA* | 0.000 | 0.000 | 0.000 | 0.000 | 0.000 | 0.000 | 0.900 | 0.652 | 0.962 |
| *DMD* | *TTN* | 0.000 | 0.000 | 0.000 | 0.000 | 0.000 | 0.000 | 0.900 | 0.868 | 0.985 |
| *STAT3* | *MAPK1* | 0.000 | 0.000 | 0.000 | 0.000 | 0.000 | 0.531 | 0.000 | 0.988 | 0.994 |
| *SOS1* | *MAP2K2* | 0.000 | 0.000 | 0.000 | 0.000 | 0.000 | 0.000 | 0.000 | 0.941 | 0.941 |
| *RAC1* | *RAF1* | 0.000 | 0.000 | 0.000 | 0.000 | 0.000 | 0.000 | 0.000 | 0.867 | 0.867 |
| *TNNI3* | *ACTC1* | 0.000 | 0.000 | 0.000 | 0.000 | 0.000 | 0.000 | 0.800 | 0.886 | 0.975 |
| *TGFB2* | *TGFB3* | 0.000 | 0.000 | 0.000 | 0.941 | 0.000 | 0.548 | 0.900 | 0.964 | 0.954 |
| *MAPK3* | *TGFB1* | 0.000 | 0.000 | 0.000 | 0.000 | 0.000 | 0.000 | 0.900 | 0.296 | 0.924 |
| *TPM3* | *MYL3* | 0.000 | 0.000 | 0.000 | 0.000 | 0.000 | 0.000 | 0.900 | 0.335 | 0.929 |
| *TTN* | *TPM4* | 0.000 | 0.000 | 0.000 | 0.000 | 0.000 | 0.000 | 0.900 | 0.000 | 0.899 |
| *TNNI3* | *MYL2* | 0.000 | 0.000 | 0.000 | 0.000 | 0.000 | 0.000 | 0.900 | 0.804 | 0.979 |
| *GRB2* | *CDC42* | 0.000 | 0.000 | 0.000 | 0.000 | 0.000 | 0.000 | 0.000 | 0.767 | 0.767 |
| *TPM4* | *MYL3* | 0.000 | 0.000 | 0.000 | 0.000 | 0.000 | 0.000 | 0.900 | 0.000 | 0.899 |
| *GSK3B* | *PXN* | 0.000 | 0.000 | 0.000 | 0.000 | 0.000 | 0.620 | 0.000 | 0.302 | 0.716 |
| *DES* | *TPM2* | 0.000 | 0.000 | 0.000 | 0.000 | 0.154 | 0.000 | 0.900 | 0.397 | 0.941 |
| *TPM2* | *TPM1* | 0.000 | 0.000 | 0.000 | 0.961 | 0.000 | 0.845 | 0.900 | 0.971 | 0.984 |
| *TNNT2* | *TNNI3* | 0.000 | 0.000 | 0.000 | 0.000 | 0.000 | 0.983 | 0.900 | 0.993 | 0.999 |
| *MYL9* | *TPM1* | 0.000 | 0.000 | 0.000 | 0.000 | 0.172 | 0.000 | 0.000 | 0.761 | 0.788 |
| *MAP2K2* | *KRAS* | 0.000 | 0.000 | 0.000 | 0.000 | 0.000 | 0.000 | 0.000 | 0.950 | 0.950 |
| *ACTN2* | *TPM2* | 0.000 | 0.000 | 0.000 | 0.000 | 0.000 | 0.000 | 0.900 | 0.537 | 0.950 |
| *DMD* | *TNNI3* | 0.000 | 0.000 | 0.000 | 0.000 | 0.000 | 0.000 | 0.900 | 0.330 | 0.928 |
| *ACTB* | *PPP1CA* | 0.000 | 0.000 | 0.000 | 0.000 | 0.000 | 0.000 | 0.900 | 0.018 | 0.899 |
| *MYH7* | *ACTC1* | 0.000 | 0.000 | 0.000 | 0.000 | 0.000 | 0.000 | 0.800 | 0.944 | 0.988 |
| *HRAS* | *BRAF* | 0.000 | 0.000 | 0.000 | 0.000 | 0.000 | 0.955 | 0.900 | 0.960 | 0.999 |
| *TPM2* | *MYL3* | 0.000 | 0.000 | 0.000 | 0.000 | 0.000 | 0.000 | 0.900 | 0.589 | 0.956 |
| *MYBPC3* | *ACTN2* | 0.000 | 0.000 | 0.000 | 0.000 | 0.000 | 0.000 | 0.900 | 0.261 | 0.921 |
| *COL2A1* | *HRAS* | 0.000 | 0.000 | 0.000 | 0.000 | 0.000 | 0.000 | 0.000 | 0.800 | 0.800 |
| *TTN* | *MYH7* | 0.000 | 0.000 | 0.000 | 0.000 | 0.331 | 0.000 | 0.000 | 0.984 | 0.989 |
| *TNNT2* | *PRKCA* | 0.000 | 0.000 | 0.000 | 0.000 | 0.000 | 0.620 | 0.000 | 0.837 | 0.934 |
| *SOS1* | *MAP2K1* | 0.000 | 0.000 | 0.000 | 0.000 | 0.000 | 0.000 | 0.900 | 0.916 | 0.991 |
| *TPM2* | *MYL2* | 0.000 | 0.000 | 0.000 | 0.000 | 0.000 | 0.000 | 0.900 | 0.215 | 0.916 |
| *MYBPC3* | *TTN* | 0.000 | 0.000 | 0.000 | 0.744 | 0.000 | 0.845 | 0.900 | 0.949 | 0.987 |
| *DMD* | *TPM1* | 0.000 | 0.000 | 0.000 | 0.000 | 0.000 | 0.000 | 0.900 | 0.766 | 0.975 |
| *ITGB1* | *PXN* | 0.000 | 0.000 | 0.000 | 0.000 | 0.000 | 0.845 | 0.800 | 0.821 | 0.993 |
| *COL2A1* | *TGFB3* | 0.000 | 0.000 | 0.000 | 0.000 | 0.000 | 0.000 | 0.000 | 0.781 | 0.780 |
| *MAP2K1* | *KRAS* | 0.000 | 0.000 | 0.000 | 0.000 | 0.000 | 0.000 | 0.900 | 0.955 | 0.995 |
| *ACTN2* | *MYL3* | 0.000 | 0.000 | 0.000 | 0.000 | 0.168 | 0.000 | 0.900 | 0.506 | 0.953 |
| *PXN* | *MAPK1* | 0.000 | 0.000 | 0.000 | 0.000 | 0.000 | 0.620 | 0.000 | 0.961 | 0.984 |
| *TPM2* | *TNNI3* | 0.000 | 0.000 | 0.000 | 0.000 | 0.000 | 0.000 | 0.900 | 0.820 | 0.980 |
| *SOS1* | *BRAF* | 0.000 | 0.000 | 0.000 | 0.000 | 0.000 | 0.000 | 0.000 | 0.950 | 0.950 |
| *PPP1CB* | *MYL9* | 0.000 | 0.000 | 0.000 | 0.000 | 0.000 | 0.000 | 0.800 | 0.407 | 0.873 |
| *MYBPC3* | *TNNT2* | 0.000 | 0.000 | 0.000 | 0.000 | 0.000 | 0.000 | 0.900 | 0.993 | 0.999 |
| *GRB2* | *HRAS* | 0.000 | 0.000 | 0.000 | 0.000 | 0.000 | 0.620 | 0.900 | 0.841 | 0.993 |
| *HRAS* | *MAP2K1* | 0.000 | 0.000 | 0.000 | 0.000 | 0.000 | 0.620 | 0.900 | 0.858 | 0.993 |
| *IGF1* | *TGFB1* | 0.000 | 0.000 | 0.000 | 0.000 | 0.000 | 0.000 | 0.000 | 0.908 | 0.908 |
| *TPM3* | *TPM4* | 0.000 | 0.000 | 0.000 | 0.966 | 0.000 | 0.000 | 0.900 | 0.951 | 0.903 |
| *PRKCA* | *TPM1* | 0.000 | 0.000 | 0.000 | 0.000 | 0.000 | 0.000 | 0.000 | 0.817 | 0.817 |
| *SOS1* | *IGF1* | 0.000 | 0.000 | 0.000 | 0.000 | 0.000 | 0.000 | 0.900 | 0.301 | 0.925 |
| *DES* | *MYL3* | 0.000 | 0.000 | 0.000 | 0.000 | 0.000 | 0.000 | 0.900 | 0.173 | 0.911 |
| *RHOA* | *ACTB* | 0.000 | 0.000 | 0.000 | 0.000 | 0.170 | 0.000 | 0.800 | 0.372 | 0.881 |
| *ACTN2* | *MYL2* | 0.000 | 0.000 | 0.000 | 0.744 | 0.186 | 0.000 | 0.900 | 0.044 | 0.913 |
| *RAC1* | *PRKCA* | 0.000 | 0.000 | 0.000 | 0.000 | 0.000 | 0.809 | 0.900 | 0.744 | 0.994 |
| *DES* | *TNNI3* | 0.000 | 0.000 | 0.000 | 0.000 | 0.000 | 0.000 | 0.900 | 0.729 | 0.971 |
| *RHEB* | *RAF1* | 0.000 | 0.000 | 0.000 | 0.000 | 0.000 | 0.986 | 0.000 | 0.493 | 0.992 |
| *HRAS* | *MAPK1* | 0.000 | 0.000 | 0.000 | 0.000 | 0.000 | 0.000 | 0.800 | 0.821 | 0.961 |
| *RAC1* | *PXN* | 0.000 | 0.000 | 0.000 | 0.000 | 0.000 | 0.000 | 0.000 | 0.786 | 0.786 |
| *MAP2K1* | *RAF1* | 0.000 | 0.000 | 0.000 | 0.643 | 0.000 | 0.993 | 0.900 | 0.992 | 0.999 |
| *COL5A1* | *TGFB1* | 0.000 | 0.000 | 0.000 | 0.000 | 0.000 | 0.000 | 0.000 | 0.858 | 0.858 |
| *TPM3* | *TPM1* | 0.000 | 0.000 | 0.286 | 0.963 | 0.000 | 0.000 | 0.900 | 0.971 | 0.904 |
| *ITGB1* | *COL5A1* | 0.000 | 0.000 | 0.000 | 0.000 | 0.000 | 0.000 | 0.800 | 0.279 | 0.846 |
| *DMD* | *MYL2* | 0.000 | 0.000 | 0.000 | 0.000 | 0.000 | 0.000 | 0.900 | 0.358 | 0.931 |
| *MAP2K1* | *MAPK1* | 0.000 | 0.000 | 0.000 | 0.652 | 0.160 | 0.998 | 0.900 | 0.994 | 0.999 |
| *TPM2* | *TTN* | 0.000 | 0.000 | 0.000 | 0.000 | 0.000 | 0.000 | 0.900 | 0.029 | 0.899 |
| *DES* | *TPM3* | 0.000 | 0.000 | 0.000 | 0.000 | 0.000 | 0.000 | 0.900 | 0.358 | 0.931 |
| *COL2A1* | *IGF1* | 0.000 | 0.000 | 0.000 | 0.000 | 0.000 | 0.000 | 0.000 | 0.953 | 0.953 |
| *ACTN2* | *TPM4* | 0.000 | 0.000 | 0.000 | 0.000 | 0.000 | 0.000 | 0.900 | 0.000 | 0.899 |
| *ACTN2* | *TNNI3* | 0.000 | 0.000 | 0.000 | 0.000 | 0.000 | 0.000 | 0.900 | 0.398 | 0.935 |
| *PRKCA* | *KRAS* | 0.000 | 0.000 | 0.000 | 0.000 | 0.000 | 0.000 | 0.800 | 0.275 | 0.845 |
| *TNNT2* | *RAC1* | 0.000 | 0.000 | 0.000 | 0.000 | 0.000 | 0.000 | 0.000 | 0.815 | 0.815 |
| *TPM2* | *TPM4* | 0.000 | 0.000 | 0.000 | 0.966 | 0.000 | 0.000 | 0.900 | 0.954 | 0.903 |
| *CDC42* | *TPM1* | 0.000 | 0.000 | 0.000 | 0.000 | 0.000 | 0.000 | 0.000 | 0.821 | 0.821 |
| *MAPK3* | *RAF1* | 0.000 | 0.000 | 0.000 | 0.658 | 0.000 | 0.812 | 0.900 | 0.989 | 0.986 |
| *ACTB* | *MYL9* | 0.000 | 0.000 | 0.000 | 0.000 | 0.000 | 0.000 | 0.800 | 0.194 | 0.827 |
| *ITGB1* | *TGFB1* | 0.000 | 0.000 | 0.000 | 0.000 | 0.000 | 0.000 | 0.000 | 0.703 | 0.703 |
| *GRB2* | *MAPK1* | 0.000 | 0.000 | 0.000 | 0.000 | 0.000 | 0.620 | 0.900 | 0.993 | 0.999 |
| *TNNT2* | *CDC42* | 0.000 | 0.000 | 0.000 | 0.000 | 0.000 | 0.000 | 0.000 | 0.815 | 0.815 |
| *MYBPC3* | *TPM4* | 0.000 | 0.000 | 0.000 | 0.000 | 0.000 | 0.000 | 0.900 | 0.000 | 0.899 |
| *TTN* | *TPM1* | 0.000 | 0.000 | 0.000 | 0.000 | 0.000 | 0.000 | 0.900 | 0.965 | 0.996 |
| *TPM2* | *ACTC1* | 0.000 | 0.000 | 0.000 | 0.000 | 0.000 | 0.000 | 0.800 | 0.199 | 0.829 |
| *DES* | *TPM1* | 0.000 | 0.000 | 0.000 | 0.000 | 0.000 | 0.000 | 0.900 | 0.829 | 0.981 |
| *TNNT2* | *ACTC1* | 0.000 | 0.000 | 0.000 | 0.000 | 0.000 | 0.000 | 0.000 | 0.936 | 0.936 |
| *RHOA* | *RAF1* | 0.000 | 0.000 | 0.000 | 0.000 | 0.000 | 0.000 | 0.000 | 0.797 | 0.797 |
| *MYH7* | *MYL3* | 0.000 | 0.000 | 0.000 | 0.000 | 0.277 | 0.000 | 0.900 | 0.922 | 0.993 |
| *BRAF* | *RAF1* | 0.000 | 0.000 | 0.000 | 0.943 | 0.000 | 0.969 | 0.900 | 0.991 | 0.996 |
| *ACTN2* | *HRAS* | 0.000 | 0.000 | 0.000 | 0.000 | 0.000 | 0.000 | 0.900 | 0.215 | 0.916 |
| *CAV1* | *TGFB1* | 0.000 | 0.000 | 0.000 | 0.000 | 0.000 | 0.000 | 0.900 | 0.239 | 0.918 |
| *MYBPC3* | *TPM1* | 0.000 | 0.000 | 0.000 | 0.000 | 0.000 | 0.000 | 0.900 | 0.941 | 0.993 |
| *TTN* | *MYL2* | 0.000 | 0.000 | 0.000 | 0.000 | 0.304 | 0.000 | 0.900 | 0.503 | 0.960 |
| *BRAF* | *KRAS* | 0.000 | 0.000 | 0.000 | 0.000 | 0.000 | 0.000 | 0.900 | 0.971 | 0.996 |
| *RHOA* | *PXN* | 0.000 | 0.000 | 0.000 | 0.000 | 0.000 | 0.000 | 0.800 | 0.820 | 0.961 |
| *DMD* | *TNNT2* | 0.000 | 0.000 | 0.000 | 0.000 | 0.000 | 0.000 | 0.900 | 0.937 | 0.993 |
| *TNNT2* | *TPM4* | 0.000 | 0.000 | 0.000 | 0.000 | 0.000 | 0.000 | 0.900 | 0.118 | 0.906 |
| *TNNI3* | *TNNC1* | 0.000 | 0.000 | 0.000 | 0.000 | 0.000 | 0.999 | 0.900 | 0.991 | 0.999 |
| *TGFB1* | *MAPK1* | 0.000 | 0.000 | 0.000 | 0.000 | 0.000 | 0.000 | 0.900 | 0.222 | 0.916 |
| *CDC42* | *PXN* | 0.000 | 0.000 | 0.000 | 0.000 | 0.000 | 0.000 | 0.000 | 0.786 | 0.786 |
| *ITGB1* | *ACTN2* | 0.000 | 0.000 | 0.000 | 0.000 | 0.000 | 0.000 | 0.800 | 0.222 | 0.833 |
| *CAV1* | *PRKCA* | 0.000 | 0.000 | 0.000 | 0.000 | 0.000 | 0.000 | 0.000 | 0.940 | 0.940 |
| *GRB2* | *RRAS* | 0.000 | 0.000 | 0.000 | 0.000 | 0.000 | 0.179 | 0.900 | 0.224 | 0.927 |
| *PRKCA* | *RAF1* | 0.000 | 0.000 | 0.000 | 0.641 | 0.000 | 0.620 | 0.900 | 0.961 | 0.973 |
| *ACTC1* | *MYL2* | 0.000 | 0.000 | 0.000 | 0.000 | 0.000 | 0.000 | 0.800 | 0.926 | 0.984 |
| *TPM4* | *TPM1* | 0.000 | 0.000 | 0.000 | 0.964 | 0.000 | 0.000 | 0.900 | 0.943 | 0.903 |
| *GRB2* | *RAF1* | 0.000 | 0.000 | 0.000 | 0.000 | 0.000 | 0.000 | 0.000 | 0.962 | 0.962 |
| *MAP2K1* | *BRAF* | 0.000 | 0.000 | 0.000 | 0.633 | 0.000 | 0.937 | 0.900 | 0.992 | 0.995 |
| *DMD* | *TPM2* | 0.000 | 0.000 | 0.000 | 0.000 | 0.000 | 0.000 | 0.900 | 0.000 | 0.899 |
| *SOS1* | *RRAS* | 0.000 | 0.000 | 0.000 | 0.000 | 0.000 | 0.620 | 0.900 | 0.398 | 0.973 |
| *DMD* | *TPM4* | 0.000 | 0.000 | 0.000 | 0.000 | 0.000 | 0.000 | 0.900 | 0.000 | 0.899 |
| *ACTN2* | *ACTC1* | 0.000 | 0.000 | 0.000 | 0.000 | 0.000 | 0.000 | 0.900 | 0.445 | 0.940 |
| *MAP2K2* | *RAF1* | 0.000 | 0.000 | 0.000 | 0.623 | 0.000 | 0.996 | 0.800 | 0.974 | 0.999 |
| *MYBPC3* | *TNNI3* | 0.000 | 0.000 | 0.000 | 0.000 | 0.000 | 0.000 | 0.900 | 0.988 | 0.998 |
| *IGF1* | *MAPK1* | 0.000 | 0.000 | 0.000 | 0.000 | 0.000 | 0.000 | 0.900 | 0.818 | 0.980 |
| *MYBPC3* | *MYL2* | 0.000 | 0.000 | 0.000 | 0.000 | 0.000 | 0.000 | 0.900 | 0.835 | 0.982 |
| *ITGB1* | *ACTB* | 0.000 | 0.000 | 0.000 | 0.000 | 0.000 | 0.000 | 0.800 | 0.188 | 0.826 |
| *DES* | *DMD* | 0.000 | 0.000 | 0.000 | 0.527 | 0.000 | 0.000 | 0.900 | 0.953 | 0.944 |
| *DES* | *TTN* | 0.000 | 0.000 | 0.000 | 0.000 | 0.000 | 0.000 | 0.900 | 0.835 | 0.982 |
| *CDC42* | *MAP2K1* | 0.000 | 0.000 | 0.000 | 0.000 | 0.000 | 0.000 | 0.000 | 0.751 | 0.751 |
| *TPM3* | *ACTN2* | 0.000 | 0.000 | 0.000 | 0.000 | 0.000 | 0.000 | 0.900 | 0.287 | 0.923 |
| *IGF1* | *COL1A2* | 0.000 | 0.000 | 0.000 | 0.000 | 0.000 | 0.000 | 0.000 | 0.950 | 0.950 |
| *ACTC1* | *TPM1* | 0.000 | 0.000 | 0.000 | 0.000 | 0.000 | 0.000 | 0.000 | 0.923 | 0.923 |
| *STAT3* | *RAF1* | 0.000 | 0.000 | 0.000 | 0.000 | 0.000 | 0.000 | 0.000 | 0.749 | 0.748 |
| *ACTN2* | *TPM1* | 0.000 | 0.000 | 0.000 | 0.000 | 0.000 | 0.000 | 0.900 | 0.564 | 0.953 |
| *MAP2K2* | *MAPK1* | 0.000 | 0.000 | 0.000 | 0.660 | 0.000 | 0.845 | 0.800 | 0.972 | 0.977 |
| *GRB2* | *MAP2K1* | 0.000 | 0.000 | 0.000 | 0.000 | 0.000 | 0.000 | 0.900 | 0.802 | 0.978 |
| *TNNT2* | *MYL9* | 0.000 | 0.000 | 0.000 | 0.000 | 0.000 | 0.000 | 0.000 | 0.725 | 0.724 |
| *RAC1* | *MAPK3* | 0.000 | 0.000 | 0.000 | 0.000 | 0.000 | 0.000 | 0.000 | 0.897 | 0.897 |
| *DMD* | *PXN* | 0.000 | 0.000 | 0.000 | 0.000 | 0.000 | 0.000 | 0.000 | 0.863 | 0.863 |
| *PPP1CA* | *MYL9* | 0.000 | 0.000 | 0.000 | 0.000 | 0.000 | 0.000 | 0.800 | 0.026 | 0.800 |
| *TTN* | *TNNI3* | 0.000 | 0.000 | 0.000 | 0.000 | 0.000 | 0.000 | 0.900 | 0.724 | 0.970 |
| *PRKCA* | *MYL9* | 0.000 | 0.000 | 0.000 | 0.000 | 0.000 | 0.000 | 0.000 | 0.836 | 0.836 |
| *TGFB2* | *COL1A2* | 0.000 | 0.000 | 0.000 | 0.000 | 0.000 | 0.000 | 0.000 | 0.949 | 0.949 |
| *MYL3* | *MYL9* | 0.000 | 0.000 | 0.000 | 0.778 | 0.000 | 0.000 | 0.900 | 0.460 | 0.909 |
| *RHOA* | *CAV1* | 0.000 | 0.000 | 0.000 | 0.000 | 0.000 | 0.620 | 0.000 | 0.739 | 0.894 |
| *SOS1* | *RAF1* | 0.000 | 0.000 | 0.000 | 0.000 | 0.000 | 0.000 | 0.000 | 0.950 | 0.950 |
| *CDC42* | *RAF1* | 0.000 | 0.000 | 0.000 | 0.000 | 0.000 | 0.000 | 0.000 | 0.953 | 0.953 |
| *MAP2K1* | *STAT3* | 0.000 | 0.000 | 0.000 | 0.000 | 0.000 | 0.000 | 0.900 | 0.781 | 0.976 |
| *PPP1CA* | *PPP1CB* | 0.000 | 0.000 | 0.525 | 0.970 | 0.000 | 0.000 | 0.900 | 0.834 | 0.903 |
| *ACTN2* | *TTN* | 0.000 | 0.000 | 0.000 | 0.000 | 0.154 | 0.809 | 0.900 | 0.863 | 0.997 |
| *MYBPC3* | *DMD* | 0.000 | 0.000 | 0.000 | 0.000 | 0.000 | 0.000 | 0.900 | 0.221 | 0.916 |
| *PPP1CB* | *MYL2* | 0.000 | 0.000 | 0.000 | 0.000 | 0.000 | 0.000 | 0.800 | 0.016 | 0.800 |
| *TPM3* | *TNNI3* | 0.000 | 0.000 | 0.000 | 0.000 | 0.000 | 0.000 | 0.900 | 0.190 | 0.913 |
| *ITGB1* | *THBS4* | 0.000 | 0.000 | 0.000 | 0.462 | 0.000 | 0.000 | 0.800 | 0.000 | 0.800 |
| *RAC1* | *CDC42* | 0.000 | 0.000 | 0.525 | 0.956 | 0.000 | 0.575 | 0.900 | 0.972 | 0.957 |
| *DMD* | *ACTB* | 0.000 | 0.000 | 0.000 | 0.000 | 0.000 | 0.000 | 0.800 | 0.341 | 0.859 |
| *TPM3* | *TPM2* | 0.000 | 0.000 | 0.000 | 0.969 | 0.000 | 0.000 | 0.900 | 0.966 | 0.902 |
| *PDGFA* | *IGF1* | 0.000 | 0.000 | 0.000 | 0.000 | 0.000 | 0.000 | 0.000 | 0.826 | 0.826 |
| *ACTN2* | *ACTB* | 0.000 | 0.000 | 0.000 | 0.000 | 0.000 | 0.900 | 0.800 | 0.318 | 0.984 |
| *ACTN2* | *DMD* | 0.000 | 0.000 | 0.000 | 0.699 | 0.000 | 0.000 | 0.900 | 0.938 | 0.928 |
| *TTN* | *TNNC1* | 0.000 | 0.000 | 0.000 | 0.000 | 0.344 | 0.000 | 0.000 | 0.796 | 0.857 |
| *GRB2* | *STAT3* | 0.000 | 0.000 | 0.000 | 0.000 | 0.000 | 0.000 | 0.900 | 0.750 | 0.973 |
| *TPM3* | *DMD* | 0.000 | 0.000 | 0.000 | 0.000 | 0.000 | 0.000 | 0.900 | 0.297 | 0.925 |
| *ACTN2* | *TGFB3* | 0.000 | 0.000 | 0.000 | 0.000 | 0.000 | 0.000 | 0.900 | 0.269 | 0.922 |
| *STAT3* | *MAPK3* | 0.000 | 0.000 | 0.000 | 0.000 | 0.000 | 0.531 | 0.000 | 0.932 | 0.966 |
| *BRAF* | *MAP2K2* | 0.000 | 0.000 | 0.000 | 0.623 | 0.000 | 0.985 | 0.800 | 0.920 | 0.998 |
| *TPM3* | *MYL2* | 0.000 | 0.000 | 0.000 | 0.000 | 0.000 | 0.000 | 0.900 | 0.270 | 0.922 |
| *TTN* | *ACTC1* | 0.000 | 0.000 | 0.000 | 0.000 | 0.000 | 0.000 | 0.000 | 0.774 | 0.774 |
| *MYL3* | *MYL2* | 0.000 | 0.000 | 0.000 | 0.788 | 0.000 | 0.000 | 0.900 | 0.965 | 0.920 |
| *RHOA* | *MYL9* | 0.000 | 0.000 | 0.000 | 0.000 | 0.000 | 0.000 | 0.800 | 0.632 | 0.921 |
| *TPM3* | *TNNT2* | 0.000 | 0.000 | 0.000 | 0.000 | 0.000 | 0.000 | 0.900 | 0.356 | 0.931 |
| *TGFB3* | *TGFB1* | 0.000 | 0.000 | 0.000 | 0.917 | 0.000 | 0.000 | 0.900 | 0.929 | 0.907 |
| *TGFB2* | *TGFB1* | 0.000 | 0.000 | 0.000 | 0.910 | 0.000 | 0.783 | 0.900 | 0.969 | 0.978 |
| *BRAF* | *PRKCA* | 0.000 | 0.000 | 0.000 | 0.625 | 0.000 | 0.559 | 0.900 | 0.000 | 0.952 |
| *COL1A2* | *TGFB1* | 0.000 | 0.000 | 0.000 | 0.000 | 0.000 | 0.000 | 0.000 | 0.977 | 0.977 |
| *PRKCA* | *RRAS* | 0.000 | 0.000 | 0.000 | 0.000 | 0.000 | 0.000 | 0.800 | 0.000 | 0.800 |
| *TNNT2* | *TPM1* | 0.000 | 0.000 | 0.000 | 0.000 | 0.000 | 0.620 | 0.900 | 0.993 | 0.999 |
| *COL2A1* | *ITGB1* | 0.000 | 0.000 | 0.000 | 0.000 | 0.000 | 0.000 | 0.900 | 0.289 | 0.924 |
| *DES* | *TNNT2* | 0.000 | 0.000 | 0.000 | 0.000 | 0.000 | 0.000 | 0.900 | 0.817 | 0.980 |
| *RAC1* | *KRAS* | 0.000 | 0.000 | 0.000 | 0.795 | 0.000 | 0.000 | 0.900 | 0.804 | 0.916 |
| *KRAS* | *RAF1* | 0.000 | 0.000 | 0.000 | 0.000 | 0.000 | 0.964 | 0.900 | 0.964 | 0.999 |
| *MYBPC3* | *MYH7* | 0.000 | 0.000 | 0.000 | 0.000 | 0.000 | 0.000 | 0.800 | 0.993 | 0.998 |
| *IGF1* | *MAPK3* | 0.000 | 0.000 | 0.000 | 0.000 | 0.000 | 0.000 | 0.900 | 0.824 | 0.981 |
| *SOS1* | *MAPK1* | 0.000 | 0.000 | 0.000 | 0.000 | 0.000 | 0.620 | 0.900 | 0.675 | 0.985 |
| *TNNT2* | *TNNC1* | 0.000 | 0.000 | 0.000 | 0.000 | 0.000 | 0.959 | 0.900 | 0.990 | 0.999 |
| *TNNI3* | *PRKCA* | 0.000 | 0.000 | 0.000 | 0.000 | 0.000 | 0.620 | 0.000 | 0.743 | 0.895 |
| *ACTB* | *MYH7* | 0.000 | 0.000 | 0.000 | 0.000 | 0.000 | 0.000 | 0.800 | 0.241 | 0.837 |
| *ITGB1* | *COL1A2* | 0.000 | 0.000 | 0.000 | 0.000 | 0.000 | 0.000 | 0.900 | 0.729 | 0.971 |
| *STAT3* | *PXN* | 0.000 | 0.000 | 0.000 | 0.000 | 0.000 | 0.000 | 0.000 | 0.760 | 0.760 |
| *TPM1* | *TNNC1* | 0.000 | 0.000 | 0.000 | 0.000 | 0.000 | 0.000 | 0.000 | 0.977 | 0.977 |
| *RAC1* | *HRAS* | 0.000 | 0.000 | 0.000 | 0.807 | 0.000 | 0.000 | 0.900 | 0.826 | 0.915 |
| *MYBPC3* | *ACTC1* | 0.000 | 0.000 | 0.000 | 0.000 | 0.000 | 0.000 | 0.000 | 0.884 | 0.884 |
| *HRAS* | *RAF1* | 0.000 | 0.000 | 0.000 | 0.000 | 0.000 | 0.999 | 0.900 | 0.987 | 0.999 |
| *PRKCA* | *PXN* | 0.000 | 0.000 | 0.000 | 0.000 | 0.000 | 0.000 | 0.800 | 0.377 | 0.867 |
| *RHOA* | *MYH7* | 0.000 | 0.000 | 0.000 | 0.000 | 0.000 | 0.000 | 0.800 | 0.566 | 0.907 |
| *TPM2* | *TNNT2* | 0.000 | 0.000 | 0.000 | 0.000 | 0.000 | 0.000 | 0.900 | 0.936 | 0.993 |
| *DES* | *MYL2* | 0.000 | 0.000 | 0.000 | 0.000 | 0.000 | 0.000 | 0.900 | 0.664 | 0.964 |
| *ACTB* | *PXN* | 0.000 | 0.000 | 0.000 | 0.000 | 0.000 | 0.000 | 0.800 | 0.412 | 0.874 |
| *HRAS* | *MAPK3* | 0.000 | 0.000 | 0.000 | 0.000 | 0.000 | 0.000 | 0.800 | 0.819 | 0.961 |
| *COL2A1* | *PRKCA* | 0.000 | 0.000 | 0.000 | 0.000 | 0.000 | 0.000 | 0.000 | 0.815 | 0.815 |
| *DMD* | *ACTC1* | 0.000 | 0.000 | 0.000 | 0.000 | 0.000 | 0.620 | 0.000 | 0.460 | 0.781 |
| *MAPK3* | *MAPK1* | 0.000 | 0.000 | 0.525 | 0.969 | 0.000 | 0.000 | 0.900 | 0.994 | 0.904 |
| *GRB2* | *PXN* | 0.000 | 0.000 | 0.000 | 0.000 | 0.000 | 0.559 | 0.000 | 0.784 | 0.898 |
| *RAC1* | *MAPK1* | 0.000 | 0.000 | 0.000 | 0.000 | 0.000 | 0.000 | 0.000 | 0.794 | 0.794 |
| *DMD* | *MYL3* | 0.000 | 0.000 | 0.000 | 0.000 | 0.000 | 0.000 | 0.900 | 0.009 | 0.899 |
| *TTN* | *MYL3* | 0.000 | 0.000 | 0.000 | 0.000 | 0.000 | 0.000 | 0.900 | 0.681 | 0.966 |
| *TPM3* | *TTN* | 0.000 | 0.000 | 0.000 | 0.000 | 0.000 | 0.000 | 0.900 | 0.005 | 0.899 |
| *ACTB* | *PPP1CB* | 0.000 | 0.000 | 0.000 | 0.000 | 0.000 | 0.000 | 0.900 | 0.044 | 0.899 |
| *ACTN2* | *TNNT2* | 0.000 | 0.000 | 0.000 | 0.000 | 0.000 | 0.000 | 0.900 | 0.631 | 0.960 |
| *RHOA* | *PRKCA* | 0.000 | 0.000 | 0.000 | 0.000 | 0.000 | 0.531 | 0.800 | 0.954 | 0.995 |
| *CDC42* | *MAPK1* | 0.000 | 0.000 | 0.000 | 0.000 | 0.000 | 0.000 | 0.000 | 0.772 | 0.772 |
| *HRAS* | *IGF1* | 0.000 | 0.000 | 0.000 | 0.000 | 0.000 | 0.000 | 0.900 | 0.309 | 0.926 |
| *TNNT2* | *MYL3* | 0.000 | 0.000 | 0.000 | 0.000 | 0.000 | 0.000 | 0.900 | 0.819 | 0.980 |
| *RAF1* | *MAPK1* | 0.000 | 0.000 | 0.000 | 0.663 | 0.000 | 0.774 | 0.900 | 0.979 | 0.983 |
| *HRAS* | *KRAS* | 0.000 | 0.000 | 0.507 | 0.968 | 0.000 | 0.000 | 0.900 | 0.972 | 0.904 |
| *GRB2* | *IGF1* | 0.000 | 0.000 | 0.000 | 0.000 | 0.000 | 0.000 | 0.900 | 0.828 | 0.981 |
| *COL2A1* | *TGFB2* | 0.000 | 0.000 | 0.000 | 0.000 | 0.000 | 0.000 | 0.000 | 0.958 | 0.958 |
| *GRB2* | *KRAS* | 0.000 | 0.000 | 0.000 | 0.000 | 0.000 | 0.000 | 0.900 | 0.724 | 0.970 |
| *RAC1* | *STAT3* | 0.000 | 0.000 | 0.000 | 0.000 | 0.000 | 0.620 | 0.900 | 0.959 | 0.998 |
| *TPM4* | *ACTC1* | 0.000 | 0.000 | 0.000 | 0.000 | 0.000 | 0.000 | 0.800 | 0.128 | 0.813 |
| *MYBPC3* | *DES* | 0.000 | 0.000 | 0.000 | 0.000 | 0.000 | 0.000 | 0.900 | 0.541 | 0.951 |
| *TGFB2* | *IGF1* | 0.000 | 0.000 | 0.000 | 0.000 | 0.000 | 0.000 | 0.000 | 0.751 | 0.750 |
| *TPM4* | *MYL2* | 0.000 | 0.000 | 0.000 | 0.000 | 0.000 | 0.000 | 0.900 | 0.000 | 0.899 |
| *MAPK3* | *PXN* | 0.000 | 0.000 | 0.000 | 0.000 | 0.000 | 0.620 | 0.000 | 0.868 | 0.946 |
| *MYH7* | *TNNI3* | 0.000 | 0.000 | 0.000 | 0.000 | 0.000 | 0.000 | 0.000 | 0.965 | 0.965 |
| *RHOA* | *MYL2* | 0.000 | 0.000 | 0.000 | 0.000 | 0.000 | 0.000 | 0.800 | 0.573 | 0.908 |
| *GRB2* | *MAPK3* | 0.000 | 0.000 | 0.000 | 0.000 | 0.000 | 0.000 | 0.900 | 0.856 | 0.984 |
| *CDC42* | *MAPK3* | 0.000 | 0.000 | 0.000 | 0.000 | 0.000 | 0.000 | 0.000 | 0.850 | 0.850 |
| *MYBPC3* | *TPM2* | 0.000 | 0.000 | 0.000 | 0.000 | 0.000 | 0.000 | 0.900 | 0.349 | 0.930 |
| *TNNI3* | *MYL3* | 0.000 | 0.000 | 0.000 | 0.000 | 0.000 | 0.000 | 0.900 | 0.825 | 0.981 |
| *PDGFA* | *TGFB1* | 0.000 | 0.000 | 0.000 | 0.000 | 0.000 | 0.000 | 0.000 | 0.815 | 0.815 |
| *TNNT2* | *MYL2* | 0.000 | 0.000 | 0.000 | 0.000 | 0.000 | 0.000 | 0.900 | 0.825 | 0.981 |
| *PPP1CA* | *MYL2* | 0.000 | 0.000 | 0.000 | 0.000 | 0.000 | 0.000 | 0.800 | 0.387 | 0.869 |
| *MYL3* | *TPM1* | 0.000 | 0.000 | 0.000 | 0.000 | 0.000 | 0.000 | 0.900 | 0.824 | 0.981 |
| *BRAF* | *RHEB* | 0.000 | 0.000 | 0.000 | 0.000 | 0.000 | 0.538 | 0.800 | 0.240 | 0.920 |
| *MYH7* | *TNNC1* | 0.000 | 0.000 | 0.000 | 0.000 | 0.458 | 0.000 | 0.000 | 0.940 | 0.965 |
| *BRAF* | *MAPK3* | 0.000 | 0.000 | 0.000 | 0.645 | 0.000 | 0.620 | 0.900 | 0.717 | 0.969 |
| *RAC1* | *MAP2K1* | 0.000 | 0.000 | 0.000 | 0.000 | 0.000 | 0.000 | 0.000 | 0.814 | 0.814 |
| *ITGB1* | *RAC1* | 0.000 | 0.000 | 0.000 | 0.000 | 0.000 | 0.000 | 0.900 | 0.293 | 0.924 |
| *MYBPC3* | *TPM3* | 0.000 | 0.000 | 0.000 | 0.000 | 0.000 | 0.000 | 0.900 | 0.000 | 0.899 |
| *BRAF* | *MAPK1* | 0.000 | 0.000 | 0.000 | 0.658 | 0.000 | 0.620 | 0.900 | 0.669 | 0.968 |
| *MYL3* | *ACTC1* | 0.000 | 0.000 | 0.000 | 0.000 | 0.000 | 0.000 | 0.800 | 0.856 | 0.969 |
| *RHOA* | *MAP2K1* | 0.000 | 0.000 | 0.000 | 0.000 | 0.000 | 0.000 | 0.000 | 0.744 | 0.744 |
| *IGF1* | *MAP2K1* | 0.000 | 0.000 | 0.000 | 0.000 | 0.000 | 0.000 | 0.000 | 0.719 | 0.719 |
| *ITGB1* | *CAV1* | 0.000 | 0.000 | 0.000 | 0.000 | 0.000 | 0.000 | 0.800 | 0.166 | 0.822 |
| *ACTC1* | *TNNC1* | 0.000 | 0.000 | 0.000 | 0.000 | 0.000 | 0.000 | 0.000 | 0.870 | 0.870 |
| *IGF1* | *STAT3* | 0.000 | 0.000 | 0.000 | 0.000 | 0.000 | 0.000 | 0.000 | 0.776 | 0.776 |
| *RHOA* | *RAC1* | 0.000 | 0.000 | 0.251 | 0.937 | 0.171 | 0.000 | 0.800 | 0.972 | 0.835 |
| *TPM1* | *MYL2* | 0.000 | 0.000 | 0.000 | 0.000 | 0.000 | 0.000 | 0.900 | 0.822 | 0.981 |
| *RHOA* | *MAPK1* | 0.000 | 0.000 | 0.000 | 0.000 | 0.000 | 0.000 | 0.000 | 0.733 | 0.733 |
| *MYH7* | *MYL2* | 0.000 | 0.000 | 0.000 | 0.000 | 0.608 | 0.000 | 0.900 | 0.887 | 0.994 |
| *MYBPC3* | *TNNC1* | 0.000 | 0.000 | 0.000 | 0.000 | 0.000 | 0.000 | 0.000 | 0.957 | 0.957 |
| *RAC1* | *GRB2* | 0.000 | 0.000 | 0.000 | 0.000 | 0.000 | 0.000 | 0.000 | 0.729 | 0.729 |
| *ACTB* | *MYL2* | 0.000 | 0.000 | 0.000 | 0.000 | 0.000 | 0.000 | 0.800 | 0.193 | 0.827 |
| *ACTB* | *PRKCA* | 0.000 | 0.000 | 0.000 | 0.000 | 0.000 | 0.000 | 0.800 | 0.440 | 0.880 |
| *ITGB1* | *PRKCA* | 0.000 | 0.000 | 0.000 | 0.000 | 0.000 | 0.845 | 0.800 | 0.097 | 0.968 |
| *DES* | *TPM4* | 0.000 | 0.000 | 0.000 | 0.000 | 0.000 | 0.000 | 0.900 | 0.202 | 0.914 |
| *TPM4* | *TNNI3* | 0.000 | 0.000 | 0.000 | 0.000 | 0.000 | 0.000 | 0.900 | 0.248 | 0.919 |
| *RAC1* | *RRAS* | 0.000 | 0.000 | 0.000 | 0.768 | 0.000 | 0.000 | 0.900 | 0.312 | 0.906 |
| *MYBPC3* | *MYL3* | 0.000 | 0.000 | 0.000 | 0.000 | 0.000 | 0.000 | 0.900 | 0.836 | 0.982 |
| *TNNT2* | *TTN* | 0.000 | 0.000 | 0.000 | 0.000 | 0.000 | 0.000 | 0.900 | 0.879 | 0.987 |
| *RAF1* | *RRAS* | 0.000 | 0.000 | 0.000 | 0.000 | 0.000 | 0.789 | 0.900 | 0.400 | 0.985 |
| *GSK3B* | *IGF1* | 0.000 | 0.000 | 0.000 | 0.000 | 0.000 | 0.000 | 0.000 | 0.787 | 0.787 |
| *MAPK3* | *KRAS* | 0.000 | 0.000 | 0.000 | 0.000 | 0.000 | 0.000 | 0.800 | 0.827 | 0.963 |
| *TGFB2* | *ACTN2* | 0.000 | 0.000 | 0.000 | 0.000 | 0.000 | 0.000 | 0.900 | 0.000 | 0.899 |
| *BRAF* | *RRAS* | 0.000 | 0.000 | 0.000 | 0.000 | 0.000 | 0.000 | 0.800 | 0.152 | 0.818 |
| *PRKCA* | *MAPK1* | 0.000 | 0.000 | 0.000 | 0.654 | 0.000 | 0.000 | 0.900 | 0.784 | 0.926 |
| *CAV1* | *COL1A2* | 0.000 | 0.000 | 0.000 | 0.000 | 0.000 | 0.000 | 0.000 | 0.849 | 0.849 |
